# Metabolic Reprogramming of NK Cells by Black Phosphorus Quantum Dots Potentiates Cancer Immunotherapy

**DOI:** 10.1002/advs.202202519

**Published:** 2023-01-22

**Authors:** Lizhen He, Jianfu Zhao, Hongjun Li, Bin Xie, Ligeng Xu, Guanning Huang, Ting Liu, Zhen Gu, Tianfeng Chen

**Affiliations:** ^1^ Department of Oncology The First Affiliated Hospital Jinan University Guangzhou 510632 P. R. China; ^2^ Key Laboratory of Advanced Drug Delivery Systems of Zhejiang Province, College of Pharmaceutical Sciences Zhejiang University Zhejiang 310000 P. R. China; ^3^ Liangzhu Laboratory Zhejiang University Medical Center Hangzhou 311121 China

**Keywords:** black phosphorus, cell metabolism, immunological enhancement, immunotherapy, natural killer cell, relieving immunosuppression

## Abstract

Low persistence, metabolic dysfunction in microenvironment, and tumor‐derived immunosuppression of Natural killer (NK) cells in patients are greatly limited the successful clinical application of NK cell‐based cancer immunotherapy. Interestingly, herein that human serum albumin‐encapsulated black phosphorus quantum dots (BPQDs@HSA) can effectively augment antitumor efficacy of clinical patients‐derived NK cell immunotherapy is found. As the donor of phosphate group, BPQDs@HSA binds with the protein of phosphatidylinositol 4‐phosphate 5‐kinase type‐1 gamma (PIP5K1A) and activates the downstream PI3K‐Akt and mTOR signaling pathways to reprogram cell metabolism of glycolysis and further promote the oxidative phosphorylation, sequentially maintains the cell viability and immunity of NK cells. And multiomics analysis is therefore conducted to reveal the underlying immunoregulation mechanisms, and that BPQDs@HSA can interact with the Toll‐like receptor (TLR) on the NK cell surface and increase the expression level of mTOR, and thus activate downstream NF‐*κ*B signalling pathways to regulate cytokine secretion and enhance immune tumoricidal is found. BPQDs@HSA can also enhance immune surveillance, relieve immune suppression, and inhibit tumor immune escape. Collectively, this study not only demonstrates a successful strategy for nanomedicine‐potentiated immune‐cancer therapy, but also sheds light on the understanding of interface between nanomedicine and immune cells activation.

## Graphical abstract

Herein a successful strategy for cancer treatment by nanomedicine‐potentiated NK cell immunotherapy is presented. BPQDs@HSA activates PI3K‐Akt‐mTOR signaling pathways to reprogram cell metabolism of glycolysis and further promote the oxidative phosphorylation, sequentially maintains the cell viability and immunity of NK cells. BPQDs@HSA can also enhance immune tumoricidal and immune surveillance, relieve immune suppression, and inhibit tumor immune escape.

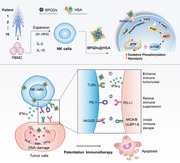

## 1 Introduction

Currently, natural killer (NK) cell therapy, a new type of cancer immunotherapy, has a unique advantage of discriminating the malignant from healthy cells, thus achieving encouraging output in pre‐clinic and clinic state research.^[^ ^1^ ^]^ According to statistics, totally, there are over two hundred of clinical trials investigating variety of NK cell immunotherapies to treat hematologic and solid malignancies in 2021.^[^ ^2^ ^]^ NK cells exert the anti‐cancer activity mainly through: 1) secreting perforin and granzymes to kill cancer cell; or 2) directly induce cell apoptosis mediated by death receptor. Another important hallmark of NK cells is the major histocompatibility complex (MHC)‐unrestricted anti‐tumor activity, which provided higher cells‐mediated immunity than other cells in cancer therapy.^[^ ^3^ ^]^ The MHC non‐restrictive characteristic makes NK cells play an important role in tumor immune surveillance, and show a significant advantage in immunotherapy to kill cancer cell more directly and rapidly.^[^ ^4^ ^]^ However, although NK cells exhibit impressive anticancer activity, the severe dysfunction, diminished activity and tumor‐derived immunosuppression of NK cells in tumor microenvironment (TME) limit the successful of cancer treatments.^[^ ^5^ ^]^ The process of tumor establishment, progression and metastasis forms an immunosuppressive microenvironment, including the highly secreted immunosuppressive factors such as TGF‐*β* and IL‐10, and the activation of PD‐1/PD‐L1 signaling pathways, to affect the immune anergy, exhaustion and cell death of NK cells or even leads to the transmission of an anti‐apoptotic signal to tumor cells. The damage of NK cells can promote the tumor establishment, progression and metastasis, and also show correlative with the prognosis of many cancer patients.^[^ ^6^ ^]^ The fundamental mechanisms of NK cell immunosuppression in TME are still poorly understood. Therefore, it is of great significance to utilize high‐efficiency and low‐toxicity sensitization agents to re‐activate and amplify NK cells activity in TME, thus enhancing their sustained anticancer activity, and further explore the potential action mechanisms.

The complexity of malignant tumors and their microenvironment makes it impossible for successful clinical treatment to be accomplished by a single technique or method, which requires scientists to continuously explore new problems that arise in the treatment process and accordingly to propose new strategies based on multimodal and integrated approaches.^[^ ^7^ ^]^ Cancer immunotherapy by utilizing nanotechnology to stimulate the immunological systems of patients to enhance anticancer effects has sparked great interest in scientists, and tremendous progress has been made in this next‐generation cancer therapeutic strategy.^[^ ^8^ ^]^ Activation of immunological systems can effectively clear residual, scattered, and dormant cancer cells in blood and lymph; stop the regeneration of cancer cells; and prevent the recurrence and metastasis of tumors. Recent studies have reported that nanomedicine‐based immunotherapy in combination with immune checkpoint inhibitors, laser and/or X‐ray can effectively enhance antitumor efficacy by stimulating the host immune system^[^ ^9^ ^]^ and regulating T cells.^[^ ^10^ ^]^ Consistently, studies also found that, targeting delivery of active molecules/cytokine by nanoparticles were able to overcome the challenges of cancer immunotherapy.^[^ ^11^ ^]^ Based on these findings, the evolving and emerging nanotechnology could provide a greater opportunity to augment immunotherapy of malignant cancers. However, the current nano‐immunotherapy only stimulates the host immune system by activating limited amounts of cytotoxic T lymphocytes (CTLs) and the tumoricidal immune cells. CTLs express the unique T cell receptor (TCR) in the surface to recognize and combine with the specific target antigen of tumor cells, which appear as the MHC‐restricted tumor‐killing effect. For example, the CD^4+^ and CD^8+^ T cells attack tumor cells through recognizing and binding with the MHC‐II and MHC‐I class molecules on the surface of targeted cells, respectively. While, tumor cells can evade immune system recognition and attack through various mechanisms, such as downregulating the expression of MHC class molecules. While, the NK cells exhibit the MHC non‐restrictive characteristic, and kill cancer cell more directly and rapidly, which providing higher cells‐mediated immunity than CTLs in cancer therapy. Furthermore, the immune cells can also be negatively regulated by the PD‐1/PD‐L1 pathway, and easily inactivated by immunosuppressive effects in tumor environment.^[^ ^12^ ^]^ To re‐activate and amplify the effect of immune cell therapy, we recently reported that cytokine‐induced killer cells expanded using IL‐15 could be boosted by selenium‐based nanoemulsions, and were capable of eliminating established tumors in mice bearing human breast cancer through regulation of NKG2D/NKG2DL activation and modulating TGF‐*β*/TGF‐*β* RI/Smad2/3 signaling.^[^ ^13^ ^]^ These findings suggest that, strategies based on stimulating the NK cell immune response could be further improved to achieve successful cancer therapy, and the potentiation mechanisms of NK cells also need more in‐depth investigation.

**Figure 1:**
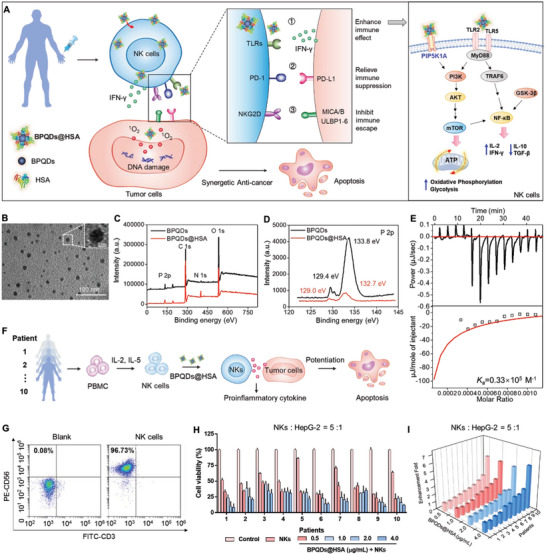
BPQDs@HSA enhances cancer‐killing activity of NK cells derived from patients (A). B) TEM image of BPQDs@HSA. XPS (C) and P 2p spectra (D) of BPQDs and BPQDs@HSA. E) ITC data for the adsorption process of HSA on BPQDs. The upper graphs exhibit the raw data obtained from the titrations, and lower graphs show the integrated energy of each peak with the corresponding fitting curve. F) BPQDs@HSA enhances cancer‐killing activity of NK cells derived from patients. G) The purity of NK cells after isolation and culture from clinical patients. H) Cell viability of HepG‐2 cells after co‐treatment with BPQDs@HSA and NK cells obtained from different patients. The ratio of NK cells to HepG‐2 cells was approximately 5:1 (means ± S.D., *n* = 3). I) Fold enhancement in anticancer activity of NK cells obtained from different patients enhanced by BPQDs@HSA. PBMC, peripheral blood mononuclear cells; NKs, NK cells.

Herein, we designed and synthesized biocompatible black phosphorus quantum dots encapsulated with human serum albumin (BPQDs@HSA) as immunosensitizers to enhance NK cell activity against tumor cells, thus achieving synergistic tumor inhibition. Black phosphorus (BP) has attracted great interest owing to its sensitization for X‐ray‐induced photodynamic therapy and excellent near‐infrared (NIR) photothermal performance.^[^ ^14^ ^]^ Furthermore, BP also exhibits high biocompatibility and biodegradability, and its metabolites are nontoxic phosphate and phosphonate.^[^ ^14b^ ^]^ In this study, we have found that BPQDs@HSA can interact with Toll‐like receptor (TLR) on the surface of NK cells and thus activate downstream NF‐*κ*B signalling pathways to regulate the secretion of cytokines by NK cells and enhance the Th1 immune response. And as the donor of phosphate group, BPQDs@HSA binds with the protein of phosphatidylinositol 4‐phosphate 5‐kinase type‐1 gamma (PIP5K1A) and activates the downstream PI3K‐Akt‐mTOR signaling pathways to reprogram cell metabolism of glycolysis and further promote the oxidative phosphorylation, sequentially maintaining the cell viability and immunity of NK cells. Examination of the mechanisms underlying the action of BPQDs@HSA as an immunosensitizer revealed that BPQDs@HSA enhances immune effects, relieves tumor‐derived immunosuppression of NK cells and inhibits the immune escape of tumor cells, leading to amplified anticancer efficacy. Multiomics analysis further revealed that, BPQDs@HSA regulated diverse effects at the individual gene and protein levels but converged to common responses at the pathway level, finally resulting in enhancement of immunocidal effects of NK cells (**Figure** **1**A). Collectively, our data contribute to a successful and translational strategy for cancer treatment by nanomedicine‐potentiated NK cell immunotherapy, and elucidate the fundamental mechanisms for regulation of cell metabolism and antitumor immunity by a BP‐based nanosystem.

## 2 Results and Discussion

### 2.1 BPQDs@HSA Reprogrammes NK Cells to Potentiate its Anticancer Action

NK cell inhibition in the TME remains a major limit to hinder NK cell immunotherapy for malignant tumors. Therefore, in this study, we constructed this BPQDs@HSA nanosystem to overcome the drawbacks of NK cells (severe dysfunction and diminished activity) to achieve synergistic cancer immunotherapy. First, ultrasmall black phosphorus quantum dots (BPQDs) were prepared by the ultra‐sonication liquid exfoliation technique as previously described.^[^ ^15^ ^]^ Here, we have developed a simple method to synthesize biocompatible nanoparticles by employing human serum albumin (HSA) to decorate isolated BPQDs and reduce the degradation of BPQDs under physiological conditions, thus increasing the stability of BPQDs. As shown in Figure 1B, BPQDs@HSA appears as highly monodisperse particles with a diameter of ≈5 ± 2 nm. The peaks at 2930 and 1650 cm^−1^ in the Raman spectra were assigned to the stretching vibration of CH3 and the *α* helical structure corresponding to amide band I of protein, suggesting successful encapsulation of BPQDs by HSA (Figure S1, Supporting Information). The presence of N in the BPQDs@HSA and the change in binding energy of P 2p in X‐ray photoelectron spectroscopy (XPS) analysis also demonstrated the successful coating of HSA on the BPQDs surface (Figure 1C,D). The results of isothermal titration calorimetry (ITC) analysis (Δ*G* < 0, Δ*H* < 0, and Δ*S* < 0) suggest that the interaction of HSA and BPQDs is mainly due to van der Waals’ forces (Figure 1E). We also explored the BPQDs stability after HSA encapsulation. As shown in Figure S2A–C, Supporting Information, when dispersed in water and exposed to air for 4 days, the color of the BPQDs solution became lighter over time, while the BPQDs@HSA solution retained color well. The corresponding UV–vis absorption spectra also demonstrate the degradation of BPQDs in aqueous solution and the high stability of BPQDs@HSA. This stable property is important to support the application of P‐based nanomaterials in NK cells potentiation under physiological conditions. Studies have found that BP is biodegradable and its degradation products are nontoxic phosphate and phosphonate.^[^ ^16^ ^]^ We then also examined the metabolites of BPQDs by ^31^P NMR spectra at different times in aqueous solution. As shown in Figure S2D, Supporting Information, after being exposed in the aqueous solution for 4 days, the characteristic absorption peaks of phosphate group appeared in supernatant of BPQDs, which can act as the donor of phosphate group to participate in phosphorylation in vivo.

To better simulate the real conditions of NK cells in patients, we isolated and expanded NK cells from ten clinically identified cancer patients (Figure 1F), and confirmed the purity of NK cells (the ratio of CD56^+^/CD3^−^ NK cells at about 96.7%, Figure 1G). By using these purified NK cells, we further confirmed the high safety of BPQDs@HSA, even at a high concentration of 10 µg mL^−1^ (Figure S3, Supporting Information). We also found that different modes of BPQDs@HSA pretreatment significantly enhanced NK cell‐induced HepG‐2 cell growth inhibition (Figure S4, Supporting Information), while free BPQDs@HSA did not inhibit HepG‐2 cell proliferation, and NK cells alone slightly inhibited HepG‐2 cell growth when applied at a 2.5:1 ratio of NK cells to HepG‐2 cells. In addition, pretreatment of NK cells with BPQDs@HSA (2 µg mL^−1^, group 7) led to a higher anticancer effect than pretreatment of HepG‐2 cells with the nanosystem (groups 4, 5, and 6), indicating a better anticancer effect from pretreatment with NK cells than from pretreatment with cancer cells. We then examined the cancer‐killing activity of NK cells from 10 patients against HepG‐2 hepatocellular carcinoma cells with BPQDs@HSA. The information of these patients and the ratio of NK cells from these patients are shown in Table S1, Supporting Information. Unexpectedly, upon stimulation by BPQDs@HSA, the NK cells from seven of ten patients demonstrated twofold higher cancer‐killing effects, and the maximum of a 6.7‐fold higher effect (Figure 1H,I and Figures S5 and S6, Supporting Information), indicating that BPQDs@HSA enhances the anticancer efficacy of NK cell immunotherapy.

### 2.2 Enhancement of Cancer Recognition Ability of NK Cells to Inhibit Immune Escape

**Figure 2:**
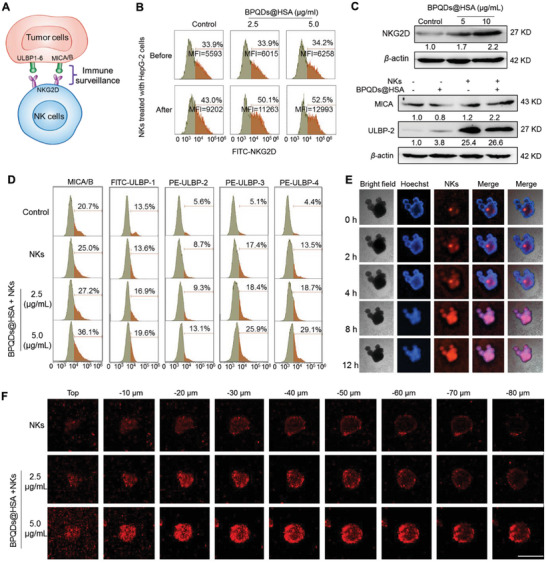
BPQDs@HSA enhances the targeting and penetration ability of NK cells against HepG‐2 cells. A) Schematic diagram of NK cells targeting tumor cells to enhance immune surveillance against tumor cells. B) Expression level of NKG2D on the NK cell surface after pre‐treated with BPQDs@HSA for 6 h and co‐incubated with HepG‐2 cells for another 24 h. NK:HepG‐2 = 5:1. C) BPQDs@HSA increases the expression of NKG2D in NK cells and the expression of MICA and ULBP‐2 in HepG‐2 cells. D) Expression level of MICA/B and ULBP 1–4 on HepG‐2 cells surface treated with BPQDs@HSA (2.5 and 5 µg mL^−1^) and NK cells (NK:HepG‐2 = 5:1). E,F) Penetration ability of NK cells into HepG‐2 tumor spheroids. NK cells were pretreated with BPQDs@HSA (5 µg mL^−1^), then labeled with CellTracker Red CMTPX Dye and visualized with red fluorescence. Scale bar, 500 µm.

NK cells can express natural killer group 2D (NKG2D) to recognize and activate its ligands, which are expressed in cancer cell membranes, including the UL16 binding protein family (ULBP 1–6) and MHC class I chain‐related molecules A and B (MICA, and MICB),^[^ ^17^ ^]^ thus enhancing the immune surveillance against tumor cells (**Figure** **2**A).^[^ ^18^ ^]^ Therefore, we examined the expression level of NKG2D on the NK cell surface after being pre‐treated with BPQDs@HSA for 6 h and co‐incubated with HepG‐2 cells for another 24 h. As shown in Figure 2B, although BPQDs@HSA didn't increase the NKG2D‐positive cell proportion in the absence of cancer cells, the mean fluorescence intensity (the value of MFI) of NKG2D expression was obviously enhanced after being treated by BPQDs@HSA. In the presence of cancer cells, NKG2D‐positive cell population and NKG2D expression level in NK cells were significantly increased, which exhibit the high cancer killing activity of these NK cells. We then also examined the NKG2D expression on the NK cell surface after incubating with BPQDs@HSA for a longer time (24 h). The results showed that the addition of BPQDs@HSA also can effectively increase the population of NKG2D‐positive cells and NKG2D expression on the NK cell surface (Figure S7, Supporting Information), suggesting the potentiation of NK cell immunotherapy by BPQDs@HSA. With Western blotting, we also examined the expression level of NKG2D in NK cells after incubation with BPQDs@HSA, and found that the NKG2D expression was also significantly increased (Figure 2C). The expression of NKG2D ligands on HepG‐2 cells was also examined after treatment with NK cells with or without BPQDs@HSA. Consistently, the expression level of MICA and ULBP‐2 ligands in HepG‐2 cells significantly increased with the presence of NK cells and BPQDs@HSA. Furthermore, as examined by fluorescence flow cytometry, BPQDs@HSA and NK cells increased the expression level of MICA/B and ULBP 1–4 on the HepG‐2 surface to varying degrees (Figure 2D). Taken together, the results indicate that upregulation of NKG2D in NK cells and its activating ligands of the MICA/B and ULBP family in cancer cells by the nanosystem could enhance the immune surveillance of tumor growth and thus prevent tumor escape from NK cell‐mediated cytotoxicity. Next, we also performed experiments to evaluate the interaction of NK cells with HepG2 cells and the ability of NK cells‐penetrated in HepG‐2 tumor spheroids, as spheroids with diameters up to 400 µm can exhibit the pathophysiological characteristics of solid tumors. As shown in Figure 2E and Video S1, Supporting Information, NK cells (red fluorescence) accumulated around HepG‐2 tumor spheroids (blue) after 2 h of treatment and then began to penetrate the tumor spheroids. After 12 h of treatment, NK cells penetrated and became dispersed in the whole tumor spheroid. We then further evaluate penetration depth of NK cells in tumor spheroids by confocal laser scanning. The fluorescence images at different layers of the spheroids from the top to the middle at −80 nm showed that BPQDs@HSA effectively potentiated the penetration ability of NK cells into HepG‐2 tumor spheroids and increased the penetration depth (Figure 2F), with a time‐dependent reduction in tumor spheroid size (Video S2, Supporting Information). Studies have found that the chemokine secreted by tumor cells can interact with chemokine receptors on the surface of NK cells, and promote NK cells infiltration into solid tumors. Therefore, we examined the expression of chemokine receptor of CXCR3 on the NK cell surface. As shown in Figure S8, Supporting Information, BPQDs@HSA can effectively increase the CXCR3 expression on the NK cell surface. Furthermore, we also examined two important chemokines secretion of CXCL9 and CXCL10 (the ligands of CXCR3) in the cultured medium of HepG‐2 cells after being incubated with BPQDs@HSA and NK cells. The results showed that the secretion of CXCL9 has no obvious change, while CXCL10 secretion in the cultured medium of HepG‐2 cells after being incubated with BPQDs@HSA and NK cells was significantly enhanced compared with the control group (Figure S9, Supporting Information). Furthermore, we also examined the CXCL10 level in the supernatant of HepG‐2 tumor spheroids, and found that BPQDs@HSA combined with NK cells effectively increased the CXCL10 secretion of tumor spheroids in a dose‐dependent manner (Figure S10, Supporting Information), which exhibited the positive correlation between the CXCL10 secretion and penetration depth of NK cells in tumor spheroids. These results indicate that the treatment of BPQDs@HSA can promote the CXCR3 expression of NK cells and interact with the enhancement secretion of CXCL10 by HepG‐2 cells to enhance the penetration of NK cells into HepG‐2 tumor spheroids. Taken together, the upregulation of NKG2D induced by BPQDs@HSA can enhance the tumor‐targeting and killing effects of NK cells, which can effectively interact with HepG‐2 cells and further enhance the penetration depth in HepG‐2 tumor spheroids. The higher cancer recognition and cancer‐penetrating ability of NK cells may increase the overall anticancer efficacy.

### 2.3 Multiomics Analysis Reveals the Mechanisms for NK Cells‐Regulation of BPQDs@HSA

High throughput multiomics approaches provide an unprecedented opportunity and technique for dissecting the molecular mechanisms accounting for immune‐regulation effects of anticancer drugs.^[^ ^19^ ^]^ Therefore, in this study, multiomics analysis was employed to explore the mechanism of action of BPQDs@HSA‐enhanced NK cell‐mediated cytotoxicity, and to discover potential therapeutic targets accounting for this effect. From the results of the transcriptomic volcano plot, 389 genes were upregulated and 302 genes were downregulated after 12‐h incubation of NK cells with BPQDs@HSA (Figure S11A, Supporting Information). In contrast, when NK cells were incubated with BPQDs@HSA for 12 h in the tumor microenvironment, the change in intracellular gene expression was significantly different, with 835 genes upregulated and 90 genes downregulated (Figure S11B, Supporting Information). Especially, the genes involved in PI3K‐AKT, mTOR, and cGMP‐PKG signaling pathway genes were significantly up‐regulated. In addition, the KEGG signaling pathway enrichment analysis reveals that, the number of genes enriched in these signaling pathways was also significantly increased (Figure S12, Supporting Information), including PI3K‐AKT, mTOR, MAPK, AMPK, TGF‐*β* and Toll‐like receptor signaling pathways, et al, which indicates that, under the tumor microenvironment, BPQDs@HSA could cause more pronounced changes in gene expression in NK cells.

**Figure 3:**
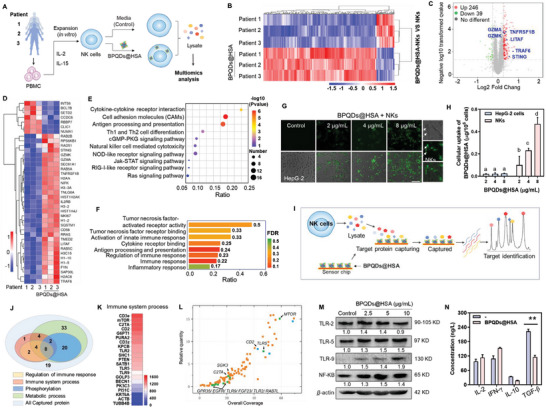
Multiomics analysis reveals the mechanisms for NK cells‐regulation effects of BPQDs@HSA. A) Schematic of multiomics analysis of NK cells derived from patients. Heatmap (B) and volcano plot (C) of the differentially expressed proteins in NK cells from three tumor patients with BPQDs@HSA incubation. D) Heatmap of immunoregulatory proteins in the three tumor patients‐donated NK cells treated by BPQDs@HSA (red indicates the increased expression and blue indicates the decreased expression). E) KEGG bubble chart of the differential proteins enrichment signaling pathway. F) Protein functional GO enrichment analysis of the differential proteins in NK cells with BPQDs@HSA treatment. The NK cells incubated with BPQDs@HSA at 5 µg mL^−1^ for 12 h were then subjected to multiomics analysis. G) Fluorescence imaging of HepG‐2 cells after treatment with NK cells for 24 h. NK cells were pretreated with FITC‐labelled BPQDs@HSA for 6 h, and the BPQDs@HSA were visualized by the green fluorescence (Scale bar, 50 µm). The white arrows indicated the NK cells. H) Quantitative analysis of the cellular uptake efficiency of BPQDs@HSA in NK cells and HepG‐2 cells (means ± S.D., *n* = 3). I) Process of BPQDs@HSA capturing the target proteins of NK cells. J) GO analysis on biological process and functions of bound proteins. K) Heatmap of the captured target proteins associated with the immune system by BPQDs@HSA. L) Scatterplots display the relative quantity of the target proteins captured by BPQDs@HSA. M) The expression levels of TLR‐2, TLR‐5, TLR‐9, and NF‐*κ*B in NK cells treated with BPQDs@HSA. N) Cytokines secretion from NK cells induced by 2 µg mL^−1^ BPQDs@HSA (means ± S.D., *n* = 3). *p < 0.05, **p < 0.01.

To understand the underlying mechanisms and potential relationship between the changes that were induced at transcriptome level and protein executors of cellular functions, we used three tumor patients‐donated NK cells to further explore the effect of BPQDs@HSA by proteomics (**Figure** **3**A). As shown in the heatmap and volcano plot of the differentially expressed proteins in NK cells (Figure 3B,C), a large number of intracellular proteins were upregulated in all the three tumor patients‐donated NK cells after treatment by BPQDs@HSA, with 246 ones upregulated and 39 ones downregulated. Among them, proteins related to NK cell immune function were significantly up‐regulated by BPQDs@HSA treatment, such as Granzyme A and K, TNF receptor‐associated factor 6 (TRAF6), tumor necrosis factor ligand superfamily member (TNLG6A, TNFRSF1B) and stimulator of interferon protein (STING). Furthermore, in these 285 changed proteins, a large number of proteins involved in NK cell viability and immune‐mediated regulation were significantly upregulated after NK cells after incubation with BPQDs@HSA for 12 h (Figure 3D). Through KEGG signaling pathway enrichment analysis (Figure 3E), these proteins participated in cytokine–cytokine receptor interaction, Th1 and Th2 cell differentiation, NK cell‐mediated cytotoxicity, and other immunoregulatory pathways. Moreover, consistent with the transcriptome, the changed proteins in NK cells after BPQDs@HSA treatment were similarly enriched to signaling pathways such as cGMP‐PKG signaling pathway, Jak‐STAT signaling pathway, and RIG‐I‐like receptor signaling pathway. Protein functional gene ontology (GO) enrichment analysis further reveals (Figure 3F) that the differential proteins after BPQDs@HSA treatment were significantly involved in tumor necrosis factor‐activated receptor activity, tumor necrosis factor receptor binding, activation of innate immune response, and cytokine receptor binding in enhancing the immunological effects of NK cells.

The treatment strategy in this study used BPQDs@HSA was as immunosensitizer to enhance NK cell immunocompetence against tumor cells. Therefore, the regulation of immune response and activation of signaling pathways in NK cells treated by BPQDs@HSA may be due to its interaction with proteins in NK cells. To further evaluate the main action of BPQDs@HSA in tumor and NK cells, we then examined the biodistribution and translocation of BPQDs@HSA in NK and HepG‐2 cells. As shown in Figure 3G,H, NK cells were pretreated with BPQDs@HSA for 12 h and then co‐incubated with HepG‐2 cells for another 24 h. It was found that most of the BPQDs@HSA remained in NK cells, and only a few BPQDs@HSA were internalized into HepG‐2 cells. These results suggest that BPQDs@HSA mainly accumulate in NK cells to regulate their immunologic function, thus enhancing anticancer activity, while the metabolites of BPQDs@HSA are phosphate and phosphonate, which are endogenous substances in vivo. Therefore, it is possible that the immune regulation mechanism is caused by the direct interaction between the complete nanoparticle of BPQDs@HSA and the proteins in NK cells. Based on these hypotheses, we employed surface plasmon resonance (SPR) technology and LC‐MS analysis to identify the protein in NK cells that was interacted by the nanosystem of BPQDs@HSA (Figure 3I). Totally, 91 proteins have been captured by BPQDs@HSA (Figure S13, Supporting Information). These bound proteins were further classified according to their biological process and functions by GO analysis. Among them, 21 proteins bound to BPQDs@HSA are involved in the immune system process, while 14 of them contribute to the regulation of immune response. 71 bound proteins are involved in the cell metabolic process and 30 ones contribute to intracellular protein phosphorylation, indicating the important roles of P element in the cell metabolism events (Figure 3J). Among the 21 proteins of the immune system process, most of them can interact strongly with BPQDs@HSA (Figure 3K). Furthermore, the scatterplot displays that the 11 bound proteins with relatively high quantity are related to the immunologic function of NK cells, especially the Toll‐like receptor, mTOR and GSK3 (Figure 3L). These results demonstrate the regulatory effects of BPQDs@HSA on the immune response of NK cells.

### 2.4 Enhancement of Immunologic Function of NK Cells by Activating TLR Pathway

Toll‐like receptor (TLR) family plays an important role in tumor immune surveillance and response by activating downstream NF‐*κ*B signalling pathways to promote pro‐inflammatory cytokine secretion and the inflammatory response. Herein, the results of target identification indicated that the Toll‐like receptor family exhibited high interaction with BPQDs@HSA, including TLR‐2, TLR‐5 and TLR‐9, so we examined the expression levels of TLR‐2, TLR‐5, TLR‐9 and NF‐*κ*B in NK cells after BPQDs@HSA treatment. As shown in Figure 3M, BPQDs@HSA obviously increased the expression levels of these proteins in NK cells, especially TLR‐5, TLR‐9 and NF‐*κ*B. This activated signalling pathway plays an important role in regulating cytokine secretion by NK cells to kill cancer cells by changing the tumor immune environment and attacking cancer cells. Therefore, we examined the levels of cytokines released from NK cells induced by BPQDs@HSA. In the supernatant of NK cells, TGF‐*β* (an important immune suppressive factor), was significantly decreased after BPQDs@HSA treatment, which could effectively relieve the immune suppression of NK cells suffered from the TME. Moreover, the expression of IFN‐*γ* and IL‐10 after being treated with BPQDs@HSA also showed an upward and downward trend, respectively (Figure 3N). Taken together, BPQDs@HSA potentiated NK cell immunotherapy mainly through relieving immune suppression, but also regulating NK cells trends to Th1 immune response, which is consistent with the result of Th1 and Th2 cell differentiation by KEGG pathway enrichment analysis of genomics and proteomics.

### 2.5 BPQDs@HSA Relieves Immune Suppression of NK Cells in TME

**Figure 4:**
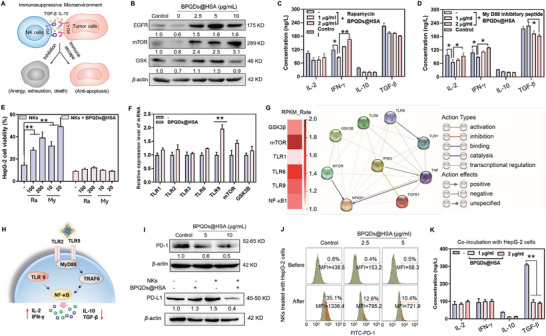
BPQDs@HSA relieves immune suppression of NK cells in TME. A) The immunosuppressive microenvironment affects the immune anergy, exhaustion, and cell death of NK cells or even leads to the transmission of an anti‐apoptotic signal to tumor cells. B) Effects of BPQDs@HSA on the expression levels of EGFR, mTOR and GSK‐3*β* in NK cells associated with cancer cell growth, proliferation and differentiation. Effects of BPQDs@HSA on the cytokine secretion from NK cells with pre‐incubation of 100 nm rapamycin (C) and 10 µm MyD88 inhibitory peptide (D) (means ± S.D., *n* = 3). E) Effects of rapamycin (Ra, 100 nm) and MyD88 inhibitory peptide (My, 10 µm) on anticancer effect of BPQDs@HSA (2 µg mL^−1^) and NK cells (NK:HepG‐2 = 5:1) (means ± S.D., *n* = 3). NK cells were pre‐treated with different concentration of these two inhibitors for 2 h. F) Effects of BPQDs@HSA (5 µg mL^−1^) on mRNA expression level of NK cells (means ± S.D., *n* = 3). *p < 0.05, **p < 0.01. G) Effects of BPQDs@HSA on related gene sequencing of NK cells. H) BPQDs@HSA enhanced the immunologic function of NK cells by activating TLR pathway. I) The expression level of PD‐1 and PD‐L1 in NK cells and HepG‐2 cells with different treatments. J) Inhibitory effects of BPQDs@HSA on the expression of PD‐1 on the surface of NK cells. NK:HepG‐2 = 5:1. K) Effects of BPQDs@HSA on the cytokine secretion from NK cells after co‐incubation with HepG‐2 cells for 24 h (means ± S.D., *n* = 3).

The process of tumor establishment, progression and metastasis forms an immunosuppressive microenvironment, including the highly secreted immunosuppressive factors such as TGF‐*β* and IL‐10, and the activation of PD‐1/PD‐L1 signalling pathways, which affects the immune anergy, exhaustion and cell death of NK cells or even leads to the transmission of an anti‐apoptotic signal to tumor cells (**Figure** **4**A).^[^ ^20^ ^]^ Interestingly, we have found that BPQDs@HSA obviously captured the proteins related to NK cell growth, proliferation and differentiation, including EGFR, mTOR and GSK‐3*β*. Although these proteins also play an important role in tumorigenesis, the treatment strategy of BPQDs@HSA was pre‐incubated with NK cells and then co‐treated with tumor cells. And from the localization of BPQDs@HSA in the co‐treatment of NK cells and HepG‐2 cells, most of the BPQDs@HSA remained in NK cells, and only a few BPQDs@HSA were internalized into HepG‐2 cells (Figure 3G,H). Therefore, we consider that BPQDs@HSA mainly accumulate in NK cells to regulate their immunologic function, thus enhancing anticancer activity. Then, we examined the expression levels of these proteins in NK cells incubated with BPQDs@HSA under TME. As shown in Figure S14, Supporting Information, the expression level of these proteins increased significantly after BPQDs@HSA treatment, indicating that BPQDs@HSA could maintain the vitality of NK cells and activate the immunologic function of NK cells. To further explore the negative effects of tumor cells against NK cells, we also examined the expression levels of EGFR, mTOR, and GSK‐3*β* in the supernatant of NK cells after co‐incubation with HepG‐2 cells by Western blotting. As shown in Figure 4B, BPQDs@HSA effectively reversed the downregulation of EGFR, mTOR and GSK‐3*β* induced by tumor cells, indicating that cancer cells may inhibit the differentiation and immune response of NK cells, but BPQDs@HSA could effectively block this negative influence. To further verify the importance of TLR pathway and mTOR pathway in the potentiation of immune response of NK cells by BPQDs@HSA, we used rapamycin (mTOR inhibitor) and MyD88 inhibitory peptide (TLR signalling inhibitor) to examine the effects of BPQDs@HSA on the cytokine secretion and anticancer activity of NK cells. As shown in Figure 4C,D, both inhibitors effectively suppressed the pro‐inflammatory cytokine (IL‐2 and IFN‐*γ*) secretion from NK cells alone, but could not affect the enhancement triggered by BPQDs@HSA. The inhibitors could also suppress the inhibitory effects of BPQDs@HSA on immune suppressive factor TGF‐*β*. Furthermore, we also examined the effects of these inhibitors on the anticancer activity of NK cells. As shown in Figure 4E, rapamycin and MyD88 inhibitory peptide at non‐toxic dose effectively suppressed the NK cells‐induced HepG‐2 cell growth inhibition, indicating that mTOR and TLR pathway play important roles in the anticancer action of NK cells. However, interestingly, the presence of BPQDs@HSA significantly stopped this effect. Furthermore, the results of polymerase chain reaction (PCR) analysis also revealed that, BPQDs@HSA treatment increased the expression of TLR‐1, ‐2, ‐3, ‐6 and ‐9, mTOR and GSK3*β* at mRNA level, which is consistent with those change induced by the nanosystem at protein level (Figure 4F). Furthermore, mRNA sequencing analysis was conducted to examine the change of NK cells. As shown in Figure 4G, treatment with BPQDs@HSA up‐regulated the gene expression levels of GSK3*β*, mTOR, TLR1, TLR6, TLR9, and NF*κ*B1. In addition, PPI was constructed by String for genes of interest, unspecific catalysis interaction was found between mTOR and NF*κ*B1, TNF and NF*κ*B1. In the TLR family, TLR1 and TLR6 are tied together in an unspecified manner. Additionally, TGF‐*β* could inhibit IFN‐*γ* expression at the transcriptional regulatory level, leading to activation of TNF secretion from NK cells. Taking the results of mRNA sequencing, captured target protein analysis and Western blotting analysis together, we can conclude that, BPQDs@HSA interact with TLR on the NK cell surface to activate downstream GSK3B‐mTOR‐NF‐*κ*B signaling pathways to regulate cytokine secretion, finally resulting in enhanced cancer therapeutic efficacy (Figure 4H).

Studies have found that the activation of PD‐1/PD‐L1 signalling pathways of NK cells in tumor immunosuppressive microenvironment is the main reason of NK cells immune dysfunction. Furthermore, the results of KEGG enrichment signalling pathway analysis showed that some of the captured target proteins by BPQDs@HSA were also enriched in the PD‐1/PD‐L1 checkpoint pathway. Therefore, we then evaluated the expression levels of PD‐1 in NK cells after treatment with BPQDs@HSA. As expected, we found that the expression levels of PD‐1 in NK cells decreased significantly after BPQDs@HSA treatment (Figure 4I). Interestingly, free NK cell treatment induced an increase in PD‐L1 expression in HepG‐2 cells. However, BPQDs@HSA‐potentiated NK cells significantly inhibited the expression of PD‐L1 in HepG‐2 cells. We then examined the expression of PD‐1 on the NK cell surface by flow cytometry. As expected, treatment with BPQDs@HSA significantly decreased the PD‐1‐positive cell population and PD‐1 expression level on the surface of NK cells in a dose‐dependent manner (Figure 4J). Interestingly, under the cancer cells‐simulated microenvironment, coincubation with HepG‐2 cells significantly enhanced the PD‐1‐positive cell population from 0.8% (control) to 35.1% to achieve immune escape, but this enhancement was also effectively inhibited by treatment of BPQDs@HSA. In addition, under the co‐incubation with HepG‐2 cell, the BPQDs@HSA‐triggered enhanced secretion of pro‐inflammatory cytokine (IL‐2 and IFN‐*γ*) from NK cells was inhibited, while still exhibiting the inhibitory effects on the secretion of immune suppressive factor IL‐10 and TGF‐*β* (Figure 4K), which further verified the remission of immune suppression effect on NK cells in a tumor microenvironment by BPQDs@HSA. These results indicate that cancer cells could activate PD‐1/PD‐L1 recognition with NK cells to cause immune anergy of NK cells, leading to the failure of immunotherapy. In contrast, BPQDs@HSA could effectively inhibit PD‐1 expression in NK cells and prevent the activation of the PD‐1/PD‐L1 pathway to relieve immune suppression of NK cells.

### 2.6 Metabolic Reprogramming of NK Cells by BPQDs@HSA

**Figure 5:**
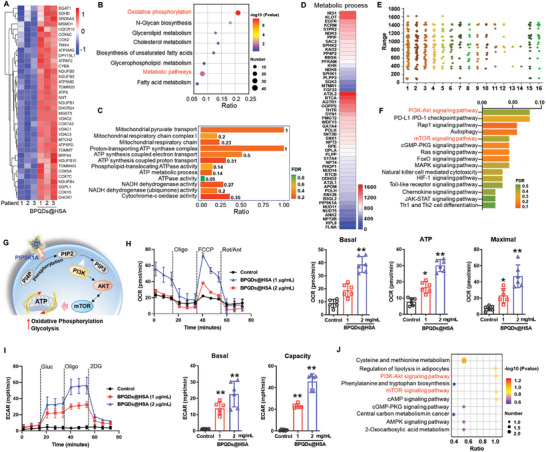
Metabolic reprogramming of NK cells by BPQDs@HSA. A) Heatmap of the proteins associated with cell metabolism in the three tumor patients‐donated NK cells treated by BPQDs@HSA (red color indicates the increased expression and blue color indicates the decreased expression). B) KEGG bubble map of differential protein enrichment in pathways related to cell metabolism. C) Protein functional GO enrichment analysis of the differential proteins associated with cellular energy metabolism. D) Heatmap of the captured target proteins associated with the cell metabolism by BPQDs@HSA. E) GO analysis on metabolic process of bound proteins (1: organic substance metabolic process, 2: organonitrogen compound metabolic process, 3: phosphorus metabolic process, 4: organophosphate metabolic process, 5: small molecule metabolic process, 6: lipid metabolic process, 7: positive regulation of macromolecule metabolic process, 8: phospholipid metabolic process, 9: glycerophospholipid metabolic process, 10: phosphatidylinositol metabolic process, 11: monosaccharide metabolic process, 12: cellular carbohydrate metabolic process, 13: pentose metabolic process, 14: D‐ribose metabolic process, 15: positive regulation of metabolic process,16: positive regulation of cellular metabolic process). F) KEGG enrichment signaling pathway of the captured target proteins by BPQDs@HSA. G) BPQDs@HSA promotes NK cell metabolism through activating PI3K‐Akt and mTOR pathway. H) OCR of NK cells treated by BPQDs@HSA for 24 h to sequential addition of Oligo, FCCP, and rotenone and antimycin (Rot/Ant). I) ECAR of NK cells treated by BPQDs@HSA for 24 h to sequential addition of glucose (Gluc), oligomycin (Oligo), and 2‐deoxy‐D‐glucose (2DG) and quantified basal glycolysis and glycolytic capacity (*p < 0.05, **p < 0.01). J) KEGG bubble map of differential metabolite‐enriched signaling pathways by metabolomics analysis.

The immunosuppressive microenvironment in solid tumors also reduces the viability of immune cells by affecting their metabolic activity, thereby inhibiting their tumor‐killing immune effects.^[^ ^21^ ^]^ Studies have found that, suppression and inhibition of NK cell anticancer action by the developed TME plays a critical role in tumor development and progression.^[^ ^5^, ^22^ ^]^ In this study, the results of proteomic analysis reveal that, BPQDs@HSA treatment upregulated most of the metabolism‐related proteins in NK cells from the three selected cancer patients (**Figure** **5**A). These differential proteins clearly participated in oxidative phosphorylation as well as lipid metabolism (Figure 5B). The results of GO enrichment analysis further reveals that a large number of proteins in NK cells after BPQDs@HSA treatment were involved in mitochondrial functions as well as ATP synthesis processes, including mitochondrial pyruvate transport, mitochondrial respiratory chain and proton‐transporting ATP synthase complex assembly, ATP synthesis coupled electron transport and NADH dehydrogenase activity (Figure 5C). These results suggest that BPQDs@HSA significantly affects the oxidative phosphorylation and glycolytic processes in NK cells. Moreover, the target‐capturing experiments also revealed that more than 70% of the intracellular proteins captured by BPQDs@HSA were involved in cellular metabolic processes, which also suggest the importance of the metabolic regulation in NK cell immunotherapy (Figure 5D). GO enrichment analysis reveals that these proteins were mainly involved in the intracellular organic substance metabolic process (66 proteins), phosphorus metabolic process (42 proteins), lipid metabolic process (21 proteins), monosaccharide metabolic process (8 proteins), and et al. Among them, 23 proteins participate in the positive regulation of metabolic process (Figure 5E). Further investigation by KEGG signaling pathway enrichment analysis of these proteins reveals that, 28% of these target proteins are involved in cellular metabolic pathways, and several other signaling pathways related to lipid metabolism and gluconeogenesis, such as glycerophospholipid metabolism, inositol phosphate metabolism, sphingolipid metabolism, pentose phosphate pathway, and fructose and mannose metabolism (Figure S15, Supporting Information). In addition, the relevant metabolic signaling pathways also point to the PI3K‐Akt signaling pathway, mTOR signaling pathways, and MAPK signaling pathway (Figure 5F). Therefore, we then examined the signaling pathway of PI3K‐Akt/mTOR in NK cells activated by BPQDs@HSA. As shown in Figure S16, Supporting Information, the expression levels of phosphoinositide 3 kinase (PI3K), AKT, and mTOR in NK cells were upregulated after treated with BPQDs@HSA. Especially, the protein of PIP5K1A was well captured by BPQDs@HSA. And as the donor of phosphate group, BPQDs@HSA binding can promote the phosphorylation‐catalyzing effect on phosphatidylinositol 4‐phosphate (PI4P) to form phosphatidylinositol 4,5‐bisphosphate (PIP2). PIP2 act as substrate to form phosphatidylinositol 3,4,5‐trisphosphate (PIP3) under the phospholipid turnover of phosphoinositide 3 kinase (PI3K), subsequently activating the downstream PI3K‐Akt‐mTOR signaling pathways to enhance the cell metabolism, especially the glycolysis (Figure 5G).^[^ ^23^ ^]^ To further evaluate the effects of BPQDs@HSA on the metabolism of NK cells, we measured the oxygen consumption rate (OCR) and extracellular acidification rate (ECAR) of NK cells incubated with BPQDs@HSA to detect their oxidative phosphorylation and glycolysis. As shown in Figure 5H, BPQDs@HSA significantly enhanced oxidative phosphorylation processes in NK cells with a dose‐dependent manner. Furthermore, we also found that the NK cells had a low basal glycolysis, after incubation with BPQDs@HSA for 24 h, the glycolysis of NK cells were significantly upregulated, which is owing to the activation of PI3K‐Akt signaling pathway by BPQDs@HSA (Figure 5I). Furthermore, the metabolomics analysis of NK cells was performed to further explore the effects of BPQDs@HSA on NK cell metabolism. From the results of the differential metabolite in NK cells (Figure S17, Supporting Information) and the KEGG bubble map of differential metabolite‐enriched signaling pathways, after BPQDs@HSA treatment, the intracellular differential metabolites could be enriched to pathways such as PI3K‐Akt and mTOR signaling pathways (Figure 5J). These results indicate that BPQDs@HSA effectively reprogram cell metabolism of glycolysis and further promote the oxidative phosphorylation of NK cells to improve cell viability and activates through PI3K‐Akt and mTOR axis to enhance the immunocidal function of NK cells.

### 2.7 X‐Ray‐Assisted Enhancement of Infiltration and Cancer‐Killing Activity of NK Cells

**Figure 6:**
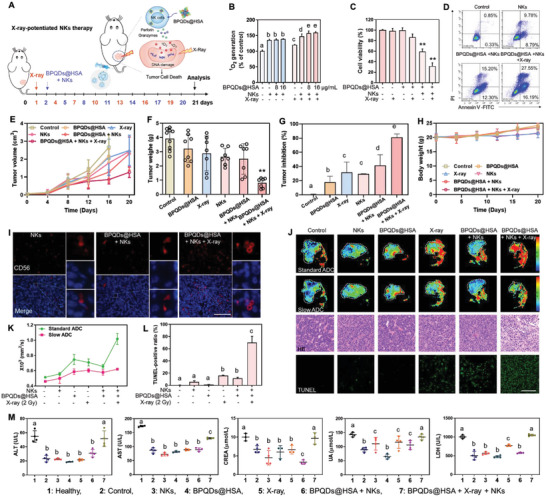
X‐ray assists BPQDs@HSA‐potentiated NK cell to penetrate into solid tumor and enhance in vivo anticancer efficacy (A). B) BPQDs@HSA‐potentiated NK cell therapy increases X‐ray‐induced ^1^O_2_ overproduction in HepG‐2 cells (means ± S.D., *n* = 3). C) X‐ray enhanced anticancer activity of BPQDs@HSA and NK cells (means ± S.D., *n* = 3). The cells were treated with 2 µg mL^−1^ of BPQDs@HSA or X‐ray (2 Gy) and NK cells (NK:HepG‐2 = 2.5:1) (*p < 0.05, **p < 0.01). D) Annexin V‐FITC/PI double staining to evaluate the HepG‐2 cell apoptosis and necrosis induced by BPQDs@HSA (2 µg mL^−1^) combined with NK cells (NK:HepG‐2 = 2.5:1) and X‐ray assistance (2 Gy). Changes in tumor volume (E), tumor weight (F), tumor inhibition (G), and body weight (H) of HepG‐2 xenografts in nude mice after intravenous injection of NK cells pre‐incubated with BPQDs@HSA (50 µg kg^−1^) and/or subjected to X‐ray radiation (2 Gy) for 7 times within 21 days (means ± S.D., *n* = 10 mice). I) Immunostaining to evaluate CD56 expression in NK cells penetrating into solid tumor tissues. Scale bar, 100 µm. J) Pseudocolor signals of MRI, H&E staining and TUNEL‐Hoechst co‐staining assays of tumor tissues from different treatment groups. Scale bar, 100 µm. K) Quantitative analysis of IVIM‐DWI parameters of tumor tissues in different treatment groups by MRI analysis (means ± S.D., *n* = 4 mice). L) Quantitative analysis of TUNEL expression of tumor tissues in different treatment groups by ImageJ analysis (means ± S.D., *n* = 4). M) Hematological analysis of liver, kidney, and heart function in the different mouse treatment groups (means ± S.D., *n* = 4 mice). Bars with different characters are statistically different at *p* < 0.05 level.

The dysfunction of NK cells in tumor microenvironment and the difficulty to penetrate into solid tumors has been identified as barriers for NK cell immunotherapy.^[^ ^5a^ ^]^ Recent studies have showed that, low‐dose X‐ray can assist nanoscale metal–organic frameworks to elicit anticancer immune‐response, through increasing immune cell infiltration and enhancing the requirement of functional B and T cells.^[^ ^10b^ ^]^ Therefore, we may ask whether X‐ray could assist NK cell to penetrate into solid tumor and enhance the in vivo anticancer efficacy of BPQDs@HSA (**Figure** **6**A). To confirm this hypothesis, first, we examined the toxicity of BPQDs@HSA against NK cells with X‐ray, and further investigate the responsiveness of the nanoparticles to X‐rays on NK cell activity. As expected, NK cells exposed on the lower X‐ray radiation (2 Gy) didn't exhibit obvious side effects, even combined with BPQDs@HSA treatment at 10 µg mL^−1^ (Figure S18, Supporting Information). Moreover, it is interestingly found that the combined treatment with NK cells and nanoparticles triggered singlet oxygen (^1^O_2_) generation in HepG‐2 hepatocellular carcinoma cells to ≈135.9% higher than that of the BPQDs@HSA treatment alone (Figure 6B), which may be due to the release of granzyme B and perforin to promote tumor cell lysis, while pretreatment of NK cells with BPQDs@HSA only slightly increased ^1^O_2_ generation in HepG‐2 cells. However, with X‐ray assistance, ^1^O_2_ generation significantly increased by 157.3% and 159.4% after incubation with BPQDs@HSA‐potentiated NK cells. In addition, as assisted by X‐ray, BPQDs@HSA‐potentiated NK cell demonstrated much higher anticancer activity (Figure 6C). The result of Annexin V and PI co‐staining assay indicate that BPQDs@HSA‐potentiated NK cell significantly enhanced HepG‐2 cell apoptosis, which was further increased with X‐ray assistance (Figure 6D).

We then further examined the X‐ray assisted enhancement of BPQDs@HSA‐potentiated NK cell immunotherapy in vivo. As shown in Figure 6E–G, the treatment of BPQDs@HSA, X‐ray radiation and NK cells alone inhibit HepG‐2 tumor growth in vivo at 17.9%, 31.4% and 29.4%, while the combination of BPQDs@HSA and NK cells slightly increased tumor inhibition to 41.3%, demonstrating the enhancement of antitumor activity of NK cells by BPQDs@HSA alone in the solid tumors. However, X‐ray‐assisted BPQDs@HSA effectively potentiated NK cells‐induced tumor inhibition rate to 81.1%, with no obvious change observed in the body weight of each group (Figure 6H). This enhancement may be due to the improvement of deep penetration of NK cells into solid tumors assisted by X‐ray radiation. We then examined the ability of NK cells to penetrate tumor tissues by CD56 (an important marker of NK cell) immunostaining. As expected, with X‐ray assistance, BPQDs@HSA‐pretreated NK cells can penetrate more into tumor tissue, as indicated by the higher expression of CD56 marker (Figure 6I and Figure S19, Supporting Information). We also employed magnetic resonance (MR) to examine tumor necrosis in living tissue and tumor growth inhibition. As shown in Figure 6J, under X‐ray assistance, BPQDs@HSA‐potentiated NK cells significantly enhanced the tumor necrosis, as evidenced by the highest values of standard ADC and slow ADC (Figure 6K). Moreover, the results of H&E staining indicated that this combined strategy significantly decreased the nuclear abnormalities, abnormal mitoses and microvascular formation, and increased the necrosis of tumor cells. This strategy also induced typical apoptotic DNA fragmentation in cancer cells in vivo, as reflected by the strong green fluorescence in the TUNEL expression of tumor tissues (Figure 6L). Taken together, these results confirm that X‐ray could assist BPQDs@HSA‐potentiated NK cell to penetrate into solid tumor and enhance the in vivo anticancer efficacy via triggering tumor cell apoptosis.

In addition, we also examined the possible toxicity of the combined strategy in vivo. As shown in Figure S20, Supporting Information, no obvious pathological changes could be observed in the major organs (including the heart, liver, spleen, lung, and kidney) after the combined treatment. Furthermore, from the images of the H&E staining, there was no inflammatory cell infiltration in the main organs with different treatment groups, which indicate the high safety of the BPQDs@HSA‐potentiated NK cell immunotherapy. Hematological analysis was also employed to examine the effects of each treatment on the functions of the liver, kidney, and heart of nude mice. As shown in Figure 6M, the tumor‐bearing nude mice exhibited a loss of balance in liver function, as revealed by changes in various blood indexes, indicating the obvious acute toxicity of tumor formation affecting the health of nude mice. However, the combined strategy significantly alleviated these damaging effects caused by tumor formation and even restored the indexes to the levels of the healthy group. Taken together, these results further confirm the safety of X‐ray‐assisted BPQDs@HSA‐potentiated NK cell immunotherapy.

## 3 Conclusion

NK cell therapy exhibits impressive anticancer activity for high cells‐mediated immunity and low tumor immune escape, but the low persistence, metabolic dysfunction in microenvironment and tumor‐derived immunosuppression of NK cells in patients limit the successful clinical application. Therefore, it is of great significance to utilize high‐efficiency and low‐toxicity sensitization agents to activate and amplify NK cells to enhance their sustained anticancer activity. Inspired by this, herein we have synthesized biocompatible BPQDs@HSA to achieve synergistic tumor inhibition by combining BPQDs‐potentiated clinical patients‐derived NK cell immunotherapy with X‐ray radiotherapy. And as the donor of phosphate group, BPQDs@HSA binds with the protein of PIP5K1A and activates the downstream PI3K‐Akt and mTOR signaling pathways to reprogram cell metabolism of glycolysis and further promote the oxidative phosphorylation, sequentially maintains the cell viability and immunity of NK cells. Multiomics analysis was therefore conducted to reveal the underlying immunoregulation mechanisms, and we found that BPQDs@HSA could interact with the TLR on the NK cell surface and increase the expression level of mTOR, and thus activate downstream NF‐*κ*B signalling pathways to regulate cytokine secretion. Interestingly, we also found that this nanosystem could downregulate PD‐1 expression in NK cells to block PD‐1/PD‐L1 recognition, thus relieving immune suppression by tumor cells and the microenvironment. In addition, this system also upregulates NKG2D to enhance immune surveillance and inhibit tumor immune escape. Furthermore, X‐ray radiation could assist BPQDs@HSA‐potentiated NK cells to penetrate into deep solid tumors, which effectively triggered tumor cell apoptosis and enhanced the in vivo antitumor activity of BPQDs@HSA‐potentiated NK cells. Collectively, our data contribute to a successful and translational strategy for cancer treatment by nanomedicine‐potentiated NK cell immunotherapy, and elucidate the fundamental mechanisms for regulation of cell metabolism and antitumor immunity by BP‐based nanosystem. This study also presents an important scientific basis for the development of high‐efficiency and low‐toxicity nanosystem based on stimulating the NK immune response and thus to achieve successful clinical cancer therapy.

## 4 Experimental Section

### Materials and Cell Lines

The BP crystals were purchased from a commercial supplier (Smart‐Elements). *N*‐methyl‐2‐pyrrolidone (NMP) (99.5%, anhydrous) and HSA were purchased from Aladdin Chemistry Co., Ltd. Thiazolyl Blue tetrazolium bromide (MTT), propidium iodide (PI), Hoechst, 2″,7″‐dichlorodihydrofluorescein diacetate (DCF‐DA) and a bicinchoninic acid (BCA) kit were purchased from Sigma‐Aldrich. Rapamycin (Sirolimus; AY 22 989) was purchased from Med Chem Express. MyD88 inhibitory peptide (RQIKIWFQNRRMKWKK‐RDVLPGTCVNS‐NH_2_) was purchased from InvivoGen. Fluorescein isothiocyanate (FITC)‐CD3 and phycoerythrin (APC)‐CD56 were purchased from BD Biosciences. Interferon‐*γ* (IFN‐*γ*), recombinant human interleukin‐2 (rhIL‐2), and monoclonal antibody CD3 (Mab‐CD3) were purchased from Cyagen. ELISA kits, including IL‐2, IL‐10, TGF‐*β* and IFN‐*γ* kits, were obtained from Bioss Antibodies. Hepatocellular carcinoma cell line (HepG‐2) was purchased from American Type Culture Collection (Manassas, VA). The cells were cultured in DMEM with 10% fetal bovine serum, 100 units/mL penicillin, and 50 units/mL streptomycin at 37 °C in a CO_2_ incubator (95% relative humidity, 5% CO_2_).

### NK Cell Culture and Expansion

The NK cells were obtained, cultured, and expanded in the liver transplantation center of 3rd Affiliated Hospital of Sun Yat‐Sen University. Briefly, peripheral blood mononuclear cells (PBMCs) were obtained from 50 mL of a volunteer's peripheral blood by density gradient centrifugation with Ficoll. The cells were suspended in fresh NK cell medium (ALYS505NK‐AC medium, Cell Science & Technology Institute, Japanese) and adjusted to a density of 2 × 10^6^ cells/mL, then incubated for 1 day at 37 °C in CO_2_ incubator (95% relative humidity, 5% CO_2_). After that, 1000 U/mL of rhIL‐2 and 10% autologous plasma were added into the cell culture medium, and continue to incubate the NK cells for another 1 day. At the third day, 30 mL of fresh ALYS505NK‐EX medium with 1000 U/mL of rhIL‐2 and rhIL‐15 were added into the cells to incubate for two days. At the fifth day, 60 mL of fresh ALYS505NK‐EX medium (Cell Science & Technology Institute, Japanese) with 1000 U/mL of rhIL‐2 were added to the cells to incubate for another two days. During the seventh to fourteen days, double fresh serum‐free ALYS505NK‐EX medium with 1000 U/mL of rhIL‐2 was added into the cells according to the medium color change. On the 14th day, the purity of NK cells was assessed by fluorescence‐activated cell sorting (CytoFLEX S, Beckman Coulter) with anti‐CD3‐FITC (Biolegend, Catalog: 300306) and anti‐CD56‐APC (Biolegend, Catalog: 362504), and the qualified cells were harvested for later use.^[^ ^24^ ^]^

### Synthesis of BPQDs@HSA Nanoparticles

The BPQDs were synthesized according to previously described procedures.^[^ ^15b^ ^]^ For BPQDs@HSA preparation, 200 µg of BP quantum dots (in NMP) were centrifuged to remove the NMP and washed with ethanol and ultrapure water. Then, 1 mL of an aqueous HSA solution (1 mg mL^−1^) was added to the aqueous BPQD solution (200 µg mL^−1^) and stirred for 24 h. In addition, a BPQDs@HSA aqueous solution was freshly synthesized.

### Characterization of BPQDs@HSA Nanoparticles

The synthesized BPQDs and BPQDs@HSA were characterized by different microscopic and spectroscopic methods. Briefly, TEM images were taken on a FEI Tecnai G2 F30 transmission electron microscope at an acceleration voltage of 200 kV. Raman scattering was performed on Horiba LabRAM HR Evolution. XPS measurement was analyzed on Thermo Fisher ESCALAB 250Xi XPS. UV–vis absorption spectra were recorded on a Lambda 25 spectrophotometer (PerkinElmer) using QS‐grade quartz cuvettes at room temperature. Isothermal titration calorimetry (ITC) (Affinity ITC, TA) was conducted at room temperature and used to examine the interaction of BPQDs and HSA.

### Cell Viability Analysis

Briefly, HepG‐2 cells (2 × 10^3^ cells/well) were seeded in 96‐well micro‐plates and attached for 24 h. The treatment modes included pre‐treatment of BPQDs@HSA with NK cells for 12 h and co‐incubation with HepG‐2 cells, administration of BPQDs@HSA and NK cells to HepG‐2 cells at the same time, and pre‐treatment of BPQDs@HSA with HepG‐2 cells for various times (2, 6, or 12 h) before administration to NK cells for another 24 h. In addition, the free BPQDs@HSA was applied to HepG‐2 cells under dark conditions for 24 h. The concentration of BPQDs@HSA in this experiment was 2 µg mL^−1^, and the NK cells were added to HepG‐2 cells at a ratio of 2.5:1 or 5:1. After incubation, the NK cells were removed and washed three times with PBS. Then, HepG‐2 cell viability was evaluated by the MTT assay as previously reported.

### Interaction of BPQDs@HSA with NK Cells and Identification Captured Proteins

To further explore the interaction between BPQDs@HSA and NK cells, surface plasmon resonance (SPR) technology using a bScreen LB 991 Label‐free Microarray System (BERTHOLD TECHNOLOGIES, Germany) and LC‐MS analysis to identify the protein in NK cells that was targeted by the nanosystem were employed. Briefly, BPQDs were suspended with ddH_2_O at 1 µg mL^−1^, and then immobilized in a sensor chip (Betterways Inc., China) with a label‐free reactive amino group modification through coordination bond. NK cell total cellular proteins were extracted by incubating cells in a lysis buffer. BPQDs were flowed through the chip surface and immobilized to the surface through coordination bond in a pH = 5.2 environment. Then NK cell lysate was flowed through chip surface. Finally, the chip was washed to remove non‐specifically attached proteins on the chip surface. The captured proteins or peptides by BPQDs were digested in situ by trypsin, and then identified with HPLC‐MS/MS. MS data were collected by Xcalibur (Thermo Scientific, version 2.2.0), and MS experiments were performed triply for each sample. The MS data were analyzed using MaxQuant software (COX LAB, version 1.3.0.5). Peptides were identified by database searching and the MS_2_ results for selected proteins that changed quantity between sample types were annotated via BLASTP. The GO analysis was carried out with Blast2GO.

### Determination of Biomarker on Surface of NK Cells

The NK cells were treated with different concentration (2.5 or 5 µg/mL) of BPQDs@HSA for 6 h. After that the cells were collected and co‐stained with anti‐CD3‐FITC (Biolegend, Catalog: 300306) and anti‐CD56‐APC (Biolegend, Catalog: 362504) according to the manufacturer's method. BPQDs@HSA treated NK cells also incubated with anti‐PD‐1 (Cell Signaling Technology) or anti‐NKG2D (Abcam) for 2 h, and then incubated with FITC‐Rabbit IgG_1_ isotype for another 0.5 h, according to the manufacturer's protocols. These biomarkers on the surface of NK cells were determined on Beckman CytoFLEX S flow cytometer.

The effects of BPQDs@HSA changing the expression of biomarker on the surface of NK cells in the tumor microenvironment were also examined with flow cytometer. NK cells were pretreated with or without BPQDs@HSA for 6 h, and then added into HepG‐2 cells at ratio of 5:1 for another 24 h. The supernatant NK cells were collected and used to determine the expression of anti‐PD‐1 and anti‐NKG2D according the methods as above. Furthermore, according to the manufacturer's method, the HepG‐2 cells were collected and respectively incubated with anti‐MICA/B (Abcam, Catalog: ab203679), anti‐ULBP1 (Abcam, Catalog: ab176566), anti‐ULBP2 (Abcam, Catalog: ab88645), anti‐ULBP3 (Abcam, Catalog: ab89931), anti‐ULBP4 (Abcam, Catalog: ab95202) for 2 h, and then respectively incubated with FITC‐Rabbit IgG1 for another 0.5 h. These biomarkers on the surface of NK cells and HepG‐2 cells were determined on Beckman CytoFLEX S flow cytometer.

### OCR and ECAR Analyses

Measurement of OCR and ECAR of NK cells were conducted by using XFp Cell Mito Stress Test Kit and XFp Glycolysis Stress Test kit on a Seahorse XF96 Extracellular Flux Analyzer (Agilent). The NK cells (2 × 10^4^/well) were seeded in metabolic 96‐wells plate, and incubated with different concentrations of BPQDs@HSA for 24 h. The reagents used in this assay were oligomycin (1.0 mm), FCCP (1.0 mm), rotenone/antimycin A (0.5 mm), glucose (10 mm), and 2‐DG (50 mm).

### Penetrating Ability of NK Cells to Tumor Spheroids

The HepG‐2 tumor spheroids were formed and grown in 6‐well plates with ultra‐low attachment surface (Corning Incorporate) according to previous procedures.^[^ ^25^ ^]^ Briefly, 1 mL of HepG‐2 cells was seeded into each well at a density of 400 000 cells/well. After tumor spheroids with diameters up to 400 µm, the tumor spheroids were removed to 35‐mm confocal dishes. Then the NK cells were labeled with Cell Tracker Red CMTPX Dye and were visualized with red fluorescence. The NK cells (1 × 10^6^ cells) were pretreated with or without BPQDs@HSA (5 µg mL^−1^) for 6 h, and then added into HepG‐2 tumor spheroids. The process of NK cells penetrated in tumor spheroids was determined by a cell imaging multi‐mode reader (Cytation 5, BioTek Instruments, Inc.). Furthermore, after NK cells were incubated with tumor spheroids for 12 h, the tumor spheroids were rinsed with PBS and scanned at the different layers from the top of the tumor spheroids to the middle using a confocal laser scanning fluorescent microscope. The video of HepG‐2 tumor spheroids attacked by BPQDs@HSA‐potentiated NK cells were determined by a cell imaging multi‐mode reader (Cytation 5, BioTek Instruments, Inc.).

### Determination of ROS Generation

The effects of BPQDs@HSA under X‐ray radiation on intracellular reactive oxygen species (ROS) generation in HepG‐2 cells (5 × 10^4^ cells/well, 96‐well micro‐plates) were examined using a DCF‐DA fluorescence probe on a micro‐plate reader (SpectraMax M5, MD, USA). In addition, BPQDs@HSA (2.5 µg mL^−1^) were applied to HepG‐2 cells for 4 h and then subjected to X‐ray radiation at 2 Gy.

The effects of drug treatment on ^1^O_2_ overproduction in HepG‐2 cells were examined by DPBF assay. The HepG‐2 cells (2 × 10^4^ cells/well, 0.1 mL) were seeded on 96‐well micro‐plates and attached for 24 h. BPQDs@HSA at 8 and 16 µg mL^−1^ incubated with NK cells for 6 h and then added in HepG‐2 cells at a ratio of 5:1, then the cells treated with or without X‐ray radiation at 2 Gy. After co‐incubation for another 12 h, the NK cells were removed and added with fresh medium, then the HepG‐2 cells were stained with the probe of DPBF (20 µm) for 1 h. The 96‐well plates were analyzed for ^1^O_2_ generation in the HepG‐2 cells using a cell imaging multi‐mode reader (Cytation 5, BioTek Instruments, Inc.) at excitation and emission wavelengths of 410 and 484 nm, respectively.

### Biodistribution and Translocation of BPQDs@HSA

The biodistribution and translocation of nanoparticles between NK and HepG‐2 cells were measured by the fluorescence intensity of FITC‐labelled BPQDs@HSA by fluorescence microscope and fluorescence microplate reader. The HepG‐2 cells were seeded into 6‐well plates at 8 × 10^4^ cells/mL (2 mL) and attached for 24 h. NK cells were pretreated with FITC‐labeled BPQDs@HSA for 6 h, and then co‐incubated with HepG‐2 cells at a ratio of 5:1 for another 24 h. The cells were then examined by fluorescence microscope, and the BPQDs@HSA was visualized by green fluorescence. Then the NK cells were removed to another blank well of the same plate and fresh medium was added, the quantitative analysis of cellular uptake efficiency of BPQDs@HSA in NK cells and HepG‐2 cells were examined by a cell imaging multi‐mode reader (Cytation 5, BioTek Instruments, Inc.).

### Flow Cytometric Analysis

Flow cytometry was employed to examine the effects of BPQDs@HSA and NK cells under X‐ray radiation on HepG‐2 cell cycle progression. Briefly, BPQDs@HSA (2 µg mL^−1^) were incubated with NK cells for 12 h and then added to HepG‐2 cells at a ratio of 2.5:1. After 24‐h incubation, the NK cells were removed and washed three times with PBS. Then, the HepG‐2 cells were collected, fixed, and stained with PI in the dark and then analyzed on a Beckman Cytomics FC500 flow cytometer. The collected cells were also stained with Annexin V‐FITC and PI according to the method in manufacturer's instructions of assay kit (Solarbio), and then analyzed on Beckman CytoFLEX S flow cytometer.

### Detection Cytokine Levels Secreted by NK Cells

One milliliter of NK cells (1 × 10^6^ cells/mL) was incubated with 2 µg mL^−1^ BPQDs@HSA for 12 h. Then, the cells were centrifuged to obtain the medium. The levels of the cytokines IL‐2, IL‐10, TGF‐*β*, and IFN‐*γ* secreted by NK cells were examined by ELISA kits (obtained from Bioss Antibodies) according to the instructions.

### Detection Chemokine Levels Secreted by HepG‐2 Cells

The HepG‐2 cells (10 × 10^4^ cells/well, 1 mL) were seeded on 24‐well micro‐plates and attached for 24 h. BPQDs@HSA at 4 µg mL^−1^ incubated with NK cells for 6 h and then added in HepG‐2 cells at ratio of 5:1. After co‐incubation for another 24 h, the supernatant medium was collected and examined the secretion of CXCL9 and CXCL10 by ELISA kits (obtained from Bioss Antibodies) according to the instructions. Furthermore, the NK cells were treated with BPQDs@HSA for 24 h, and then stained with anti‐CXCR3‐Alexa Fluor 488 (Biolegend, Catalog: 353709) according to the manufacturer's protocols.

### Western Blot Analysis

Total cellular proteins (1 × 10^5^ cells/mL, 10 mL/vessel) in the groups treated with different concentration of free BPQDs@HSA (2.5, 5.0, and 10 µg mL^−1^, in the dark) or NK cells (NK:HepG‐2 = 5:1) or co‐treated with BPQDs@HSA and NK cells were extracted by cell lysis buffer from Cell Signaling Technology. The protein concentrations were examined by the BCA protein assay. The effects of BPQDs@HSA and NK cells on the expression levels of related proteins were determined by Western blotting.

### Real‐Time PCR Analysis

Total RNA of NK cells after different treatment was extracted using TRIzol and subjected to reverse transcription with a ReverTra Ace‐a‐kit (Takara) according to the manufacturer's protocol. Real‐time PCR analysis was performed using a SYBR Green PCR Master Mix kit (Bimake) on an Opticon 2 Real Time Cycler instrument (Bio‐red, CFX Connect). The primer sequences of the related genes were listed in the Table S2, and the ΔΔCt method was used to quantify the mRNA expression level.

### mRNA Sequencing Analysis of NK Cells after Treatment

After treatment with BPQDs@HSA (5 µg mL^−1^) and X‐ray (2 Gy), RNA from the NK cells was extracted using TRIzol reagent. RNA was precipitated at −20 °C overnight. Then the mRNA library was constructed using VAHTS mRNA‐seq V3 Library Prep Kit following the manufacturer's instructions. Libraries were sequenced on an Illumina NovaSeq 6000 sequencer for 318 cycles. Adapters were trimmed from the reads, and the reads shorter than 17 nt were discarded. The reads were mapped to the human mRNA reference database on Chi‐Cloud NGS Analysis Platform (Chi‐Biotech Co. Ltd., Shenzhen, China). PPI analysis of differentially expressed genes was based on the STRING database, which known and predicted protein–protein interactions.

### In Vivo Antitumor Activity

HepG‐2 cell xenografts were established with ≈1 × 10^6^ cells in 100 µL of serum‐free medium injected into the right oxter of male BALB/c nude mice. After the tumor volume increased to ≈70 mm^3^, the mice were randomly divided into six treatment groups (*n* = 10). The radiotherapy treatment mice were conducted the tumor sites under X‐ray radiation for 2 Gy. After 24 h, the mice received caudal vein injection of the NK cells with pre‐incubation of BPQDs@HSA (50 µg kg^−1^) for 12 h. In addition, each mouse was injected with 5 × 10^5^ NK cells in 100 µL of X‐vivo medium via the tail vein twice at intervals of one week. Throughout the 3 weeks, changes in the tumor volume and body weight were monitored. On the 21st day, the tumor tissues of each treatment group were harvested and weighed. The tumor sections were subjected to H&E staining and TUNEL‐Hoechst co‐staining. The expression levels of PD‐1 and PD‐L1 protein in 2‐µm tumor sections were examined by immunohistochemical and immunofluorescence methods. Venous blood was collected from the nude mouse eyes at the end of the experiments, and hematological analysis was conducted to examine the kidney, liver and heart functions of the nude mice. Intravoxel incoherent motion diffusion‐weighted imaging (IVIM‐DWI) was performed on a 3.0‐T MR scanner (General Electric, Milwaukee, WI, USA) in living nude mice on the 21st day. The mice with tumor volume larger than 2.5 cm^3^ were regarded as dead. All of the animal studies were approved by the Animal Experimentation Ethics Committee of Jinan University (GB/T 35892‐2018).

### Statistical Analysis

The experiments were carried out at least three times and all the data were expressed as mean ± standard deviation. Statistical analysis was performed with the SPSS statistical package (SPSS 13.0 for Windows; SPSS, Inc. Chicago, IL) and differences of *p* < 0.05 (*) or *p* < 0.01 (**) were considered statistically significant.

## Conflict of Interest

The authors declare no conflict of interest.

## Supporting information

Supporting Information

Supplemental Video 1

Supplemental Video 2

## Data Availability

The data that support the findings of this study are available from the corresponding author upon reasonable request.
