# Supplementary material for: Metabolic Reprogramming of NK Cells by Black Phosphorus Quantum Dots Potentiates Cancer Immunotherapy
Source: Adv Sci (Weinh). 2023 Jan 22;10(8):2202519. doi: 10.1002/advs.202202519 (PMC10015887; doi:10.1002/advs.202202519)
Supplement: Supplementary file 1 — Supporting Information [file ADVS-10-2202519-s002.pdf]

ADVANCED SCIENCE
Open Access

Supporting Information

for *Adv. Sci.*, DOI 10.1002/advs.202202519

Metabolic Reprogramming of NK Cells by Black Phosphorus Quantum Dots Potentiates Cancer Immunotherapy

Lizhen He, Jianfu Zhao, Hongjun Li, Bin Xie, Ligeng Xu, Guanning Huang, Ting Liu, Zhen Gu and Tianfeng Chen**

Supporting Information

Metabolic reprogramming of NK cells by black phosphorus quantum dots potentiates cancer immunotherapy

Lizhen He, Jianfu Zhao, Hongjun Li, Bin Xie, Ligeng Xu, Guanning Huang, Ting Liu, Zhen Gu, Tianfeng Chen**

The results:

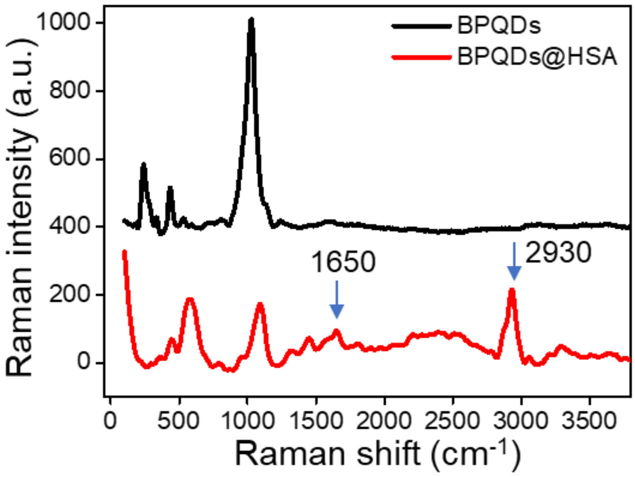

Figure S1. Raman spectra of BPQDs and BPQDs@HSA.

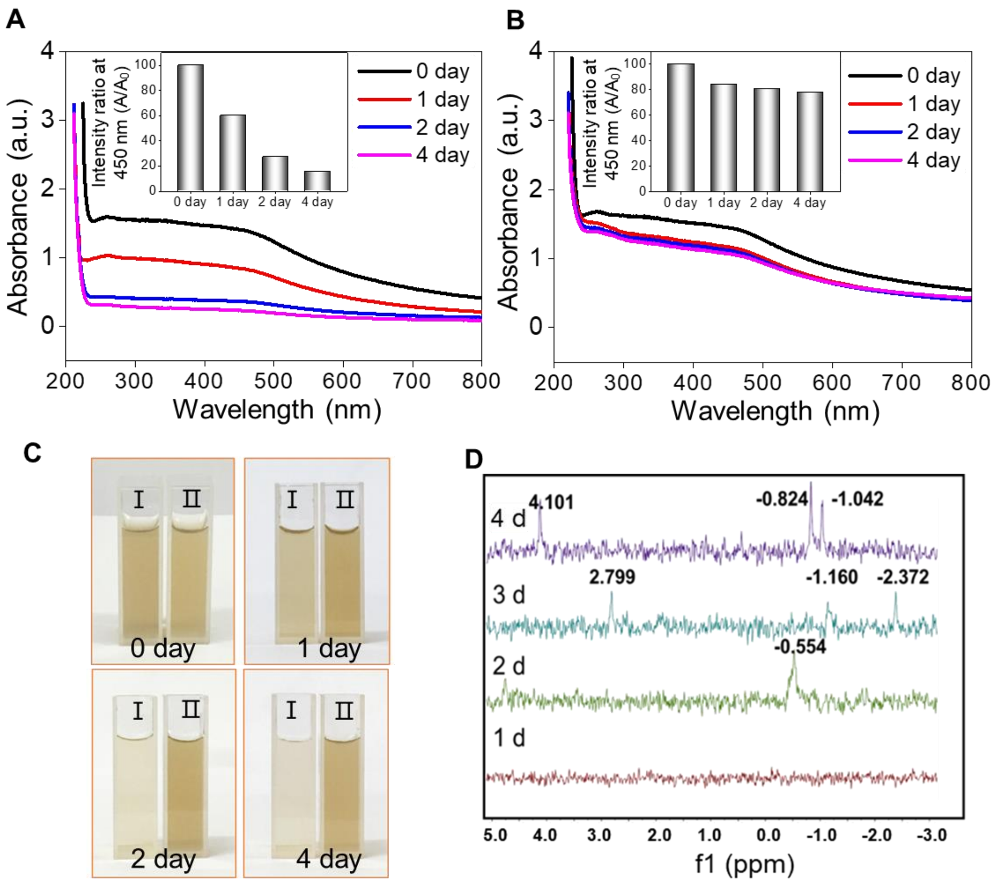

Figure S2. Stability analysis of BPQDs and BPQDs@HSA. (**A-C**) Photographs and UV-vis absorption spectra of BPQDs (I, **A**) and BPQDs@HSA (II, **B**) at different times in aqueous solution. (**D**) The ^{31}P NMR spectra of BPQDs at different times in aqueous solution.

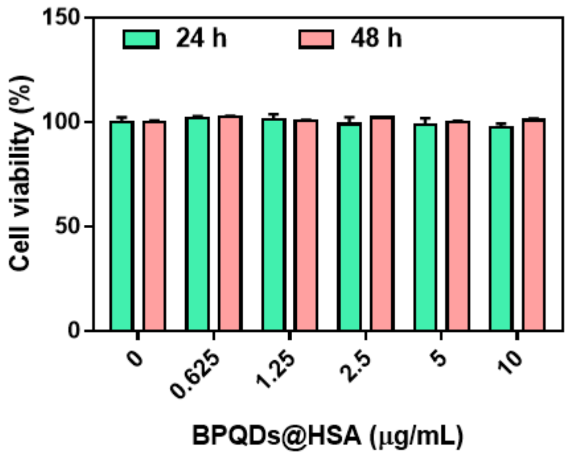

Figure S3. Cytotoxic effects of BPQDs@HSA on NK cells (means ± S.D., n = 3).

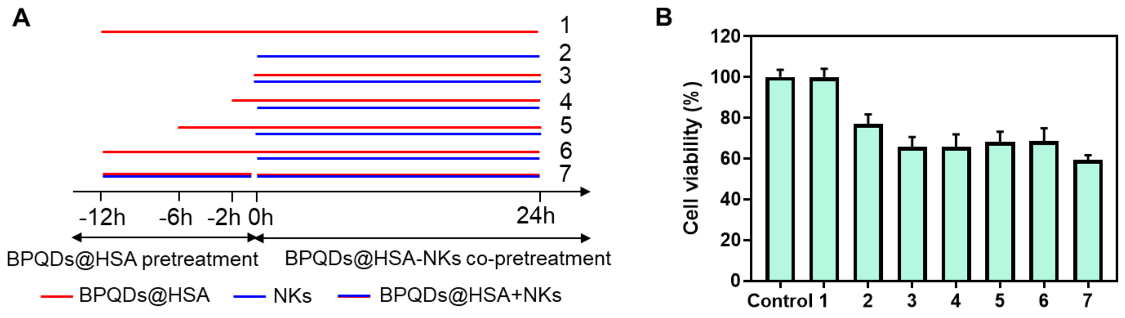

Figure S4. Optimization of the combined treatment strategy. (**A**) Combined treatment models of BPQDs@HSA with NK cells to HepG-2 cells (1: BPQDs@HSA directly incubated with HepG-2 cells for 36 h. 2: NK cells directly incubated with HepG-2 cells for 24 h. 3-6: BPQDs@HSA pretreated with HepG-2 cells for (0, 2, 6 and 12 h) and then added with NK cells for another 24 h. 7: BPQDs@HSA pretreated with NK cells for 12 h and then co-incubated with HepG-2 cells for 24 h). (**B**) Cell viability of HepG-2 cells after treated with BPQDs@HSA (2 μg/mL) and NK cells (NK: HepG-2 = 2.5: 1) (means ± S.D., n = 3).

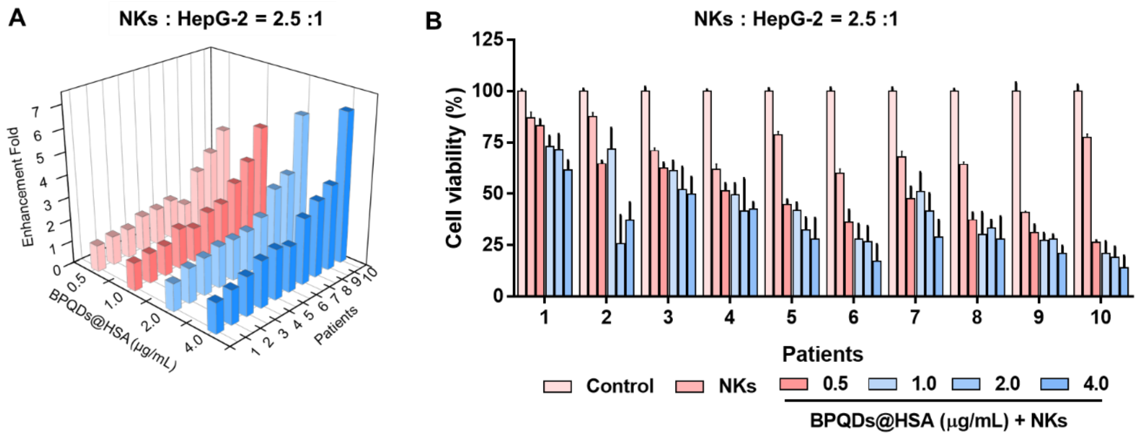

Figure S5. BPQDs@HSA enhances anticancer activity of NKs. (**A**) Fold enhancement in anticancer activity of NK cells obtained from different patients enhanced by BPQDs@HSA. (**B**) Cell viability of HepG-2 cells after co-treatment with BPQDs@HSA and NK cells obtained from different patients. The ratio of NK cells to HepG-2 cells was approximately 2.5:1 (means ± S.D., *n* = 3).

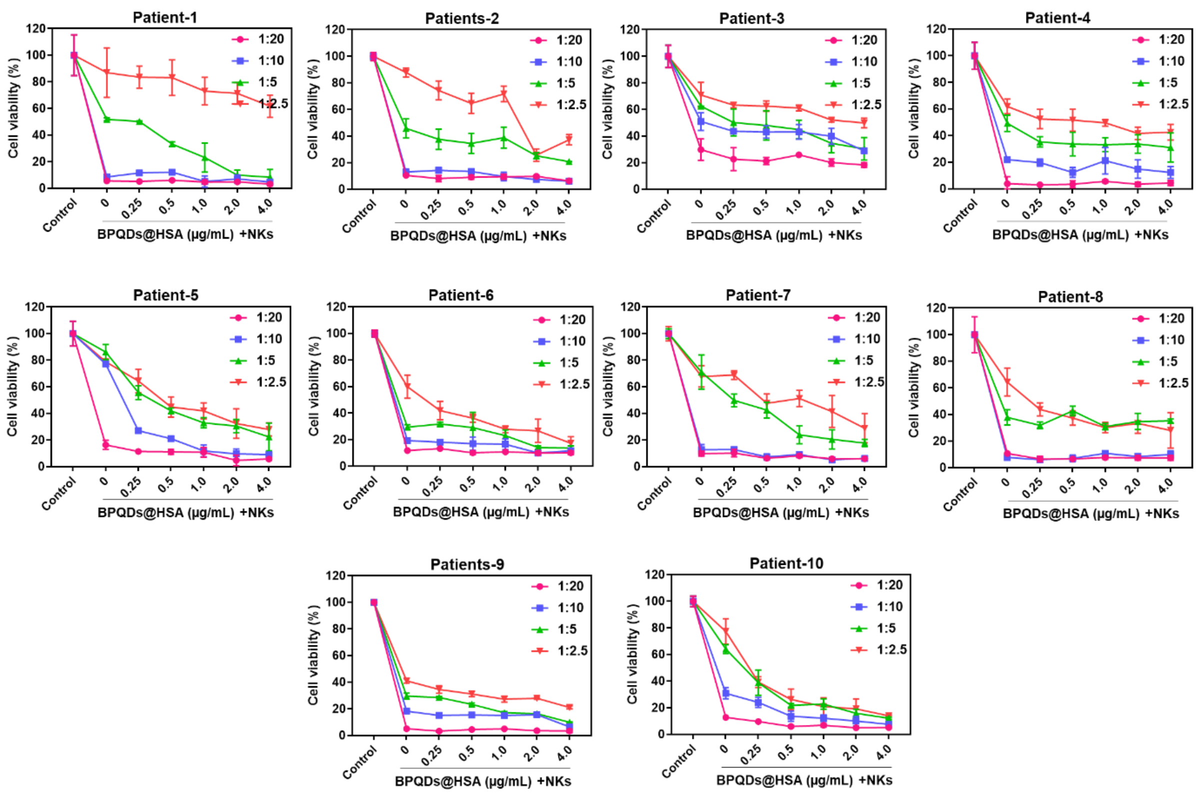

Figure S6. HepG-2 cell viability after co-treatment with BPQDs@HSA and NK cells obtained from different patients (means ± S.D., n = 3). The ratio of NK cells and HepG-2 cells was with 20:1, 10:1, 5:1 and 2.5:1.

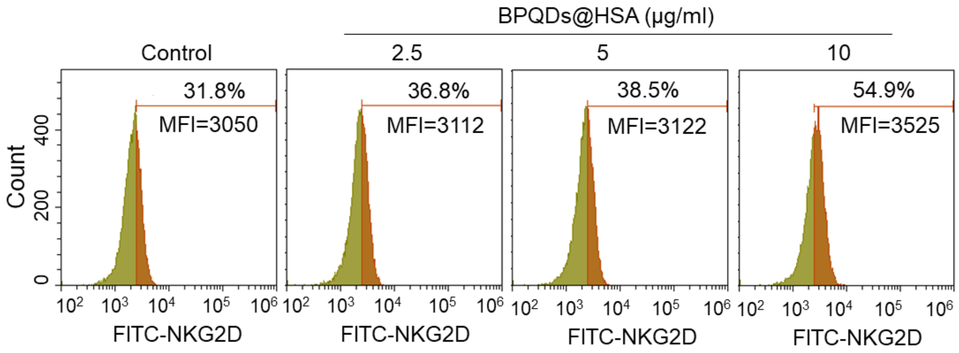

Figure S7. Expression level of NKG2D on the NK cell surface after treated with BPQDs@HSA for 24 h.

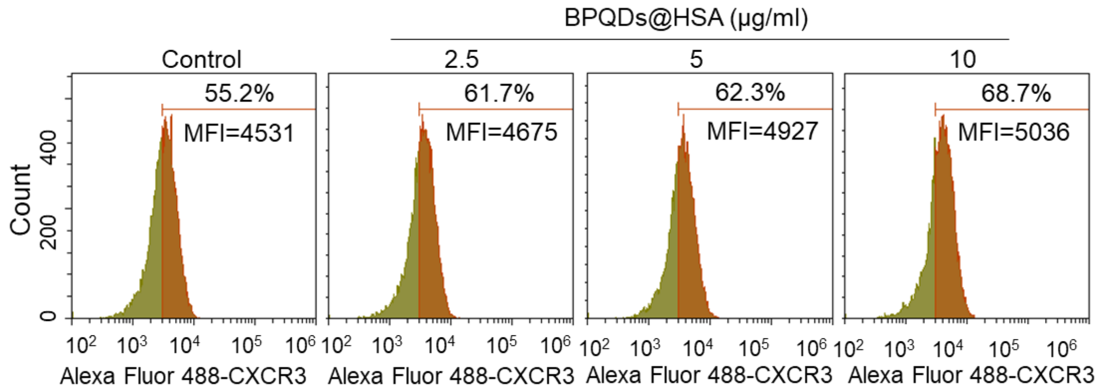

Figure S8. Expression level of CXCR3 on the NK cell surface after treated with BPQDs@HSA for 24 h.

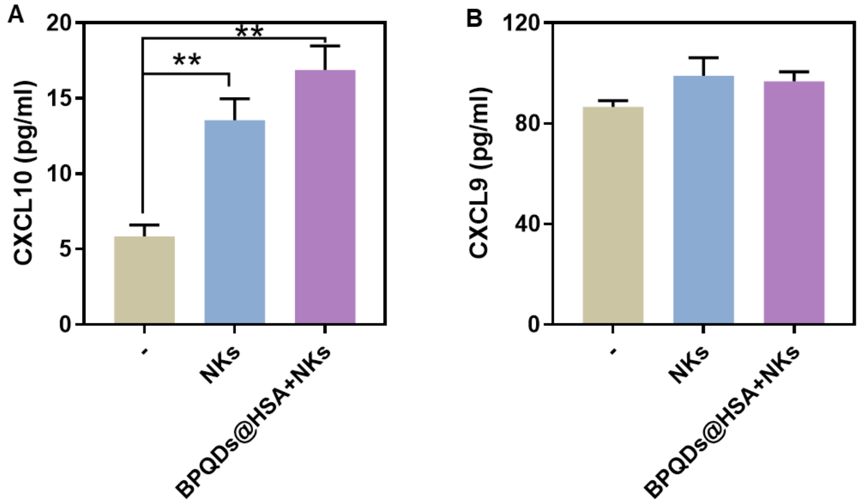

Figure S9. Chemokine secretion of CXCL10 (**A**) and CXCL9 (**B**) in the cultured medium of HepG-2 cells after incubated with BPQDs@HSA and NK cells.

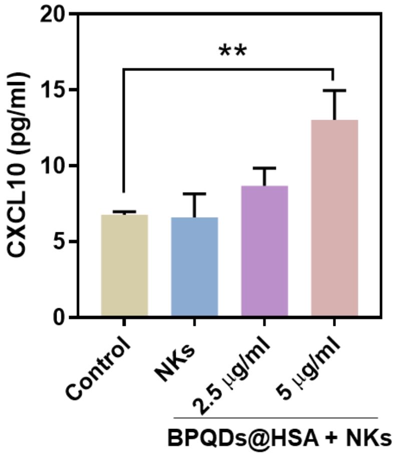

Figure S10. Chemokine secretion of CXCL10 in the supernatant of HepG-2 tumor spheroids after incubated with BPQDs@HSA.

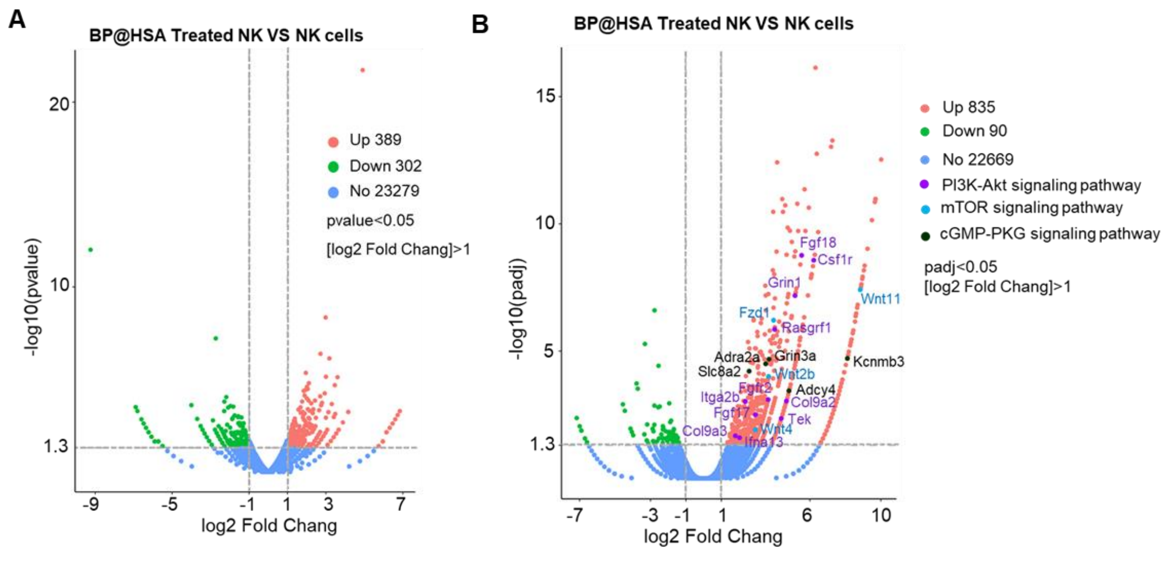

Figure S11. Vocano plot of the changed gene in the NK cells treated by BPQDs@HSA for 12 h in culture medium (**A**) and in HepG-2 cell culture medium (**B**).

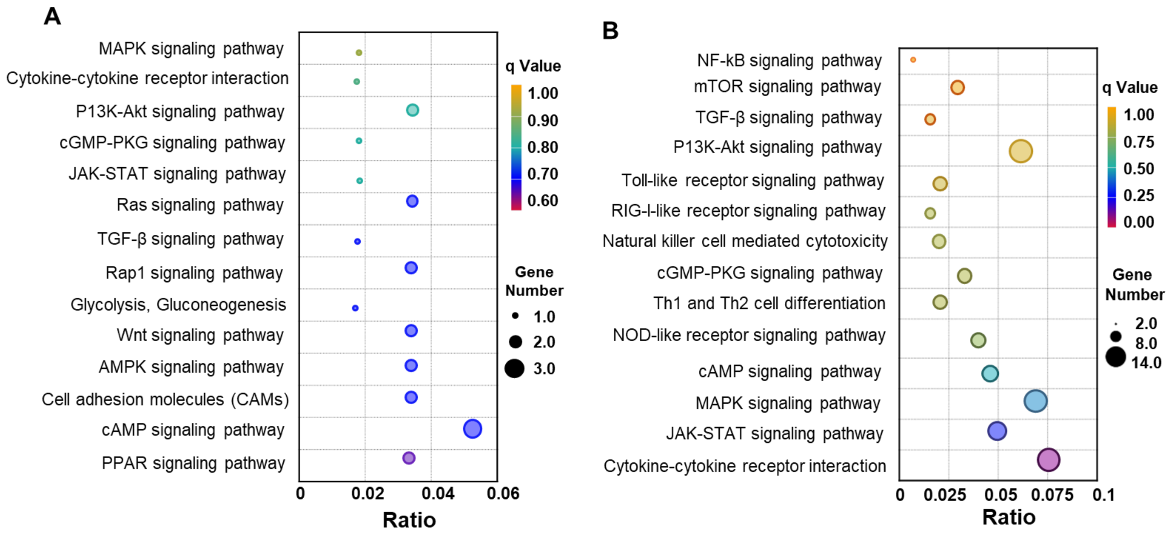

Figure S12. KEGG signaling pathway enrichment of the changed gene in the NK cells treated by BPQDs@HSA for 24 h in culture medium (**A**) and in HepG-2 cell culture medium (**B**).

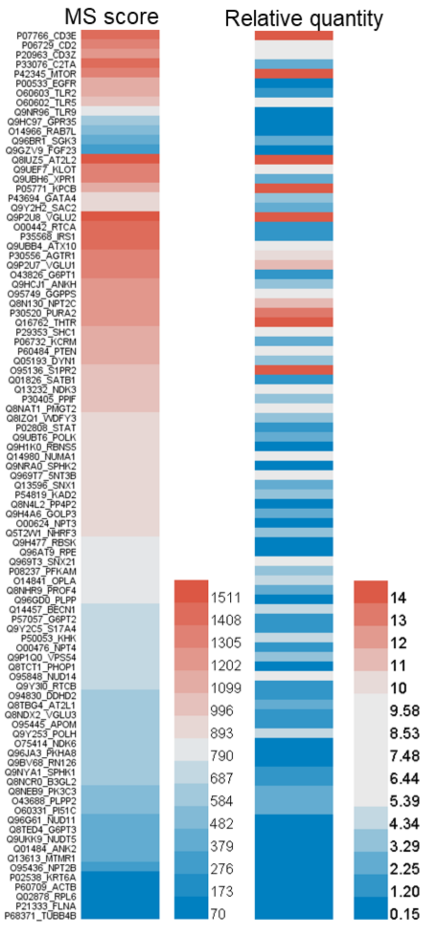

Figure S13. MS score and relative quantity of captured target proteins by BPQDs@HSA.

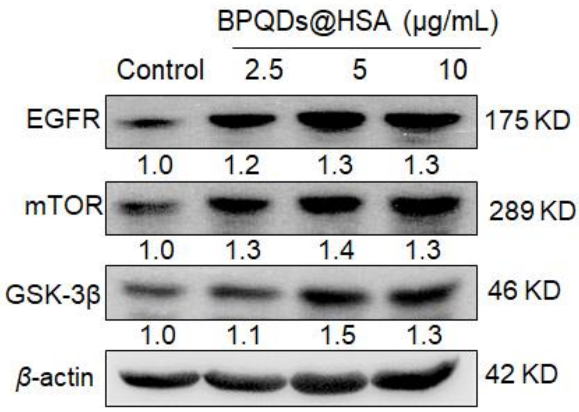

Figure S14. Expression level of EGFR, mTOR and GSK-3β in NK cells treated with BPQDs@HSA.

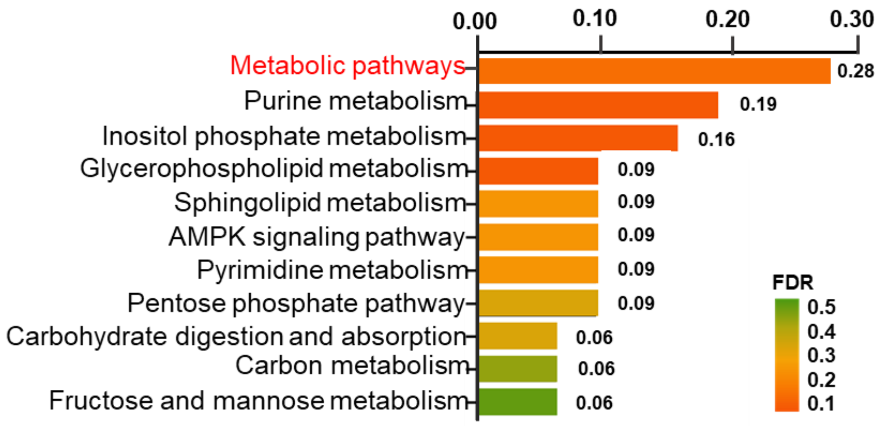

Figure S15. KEGG enrichment signaling pathway in cell metabolism of the captured target proteins by BPQDs@HSA.

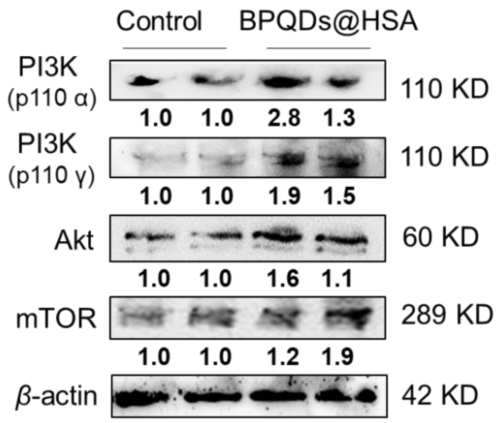

Figure S16. Expression level of PI3K, Akt and mTOR in NK cells treated with BPQDs@HSA (5 μg/mL).

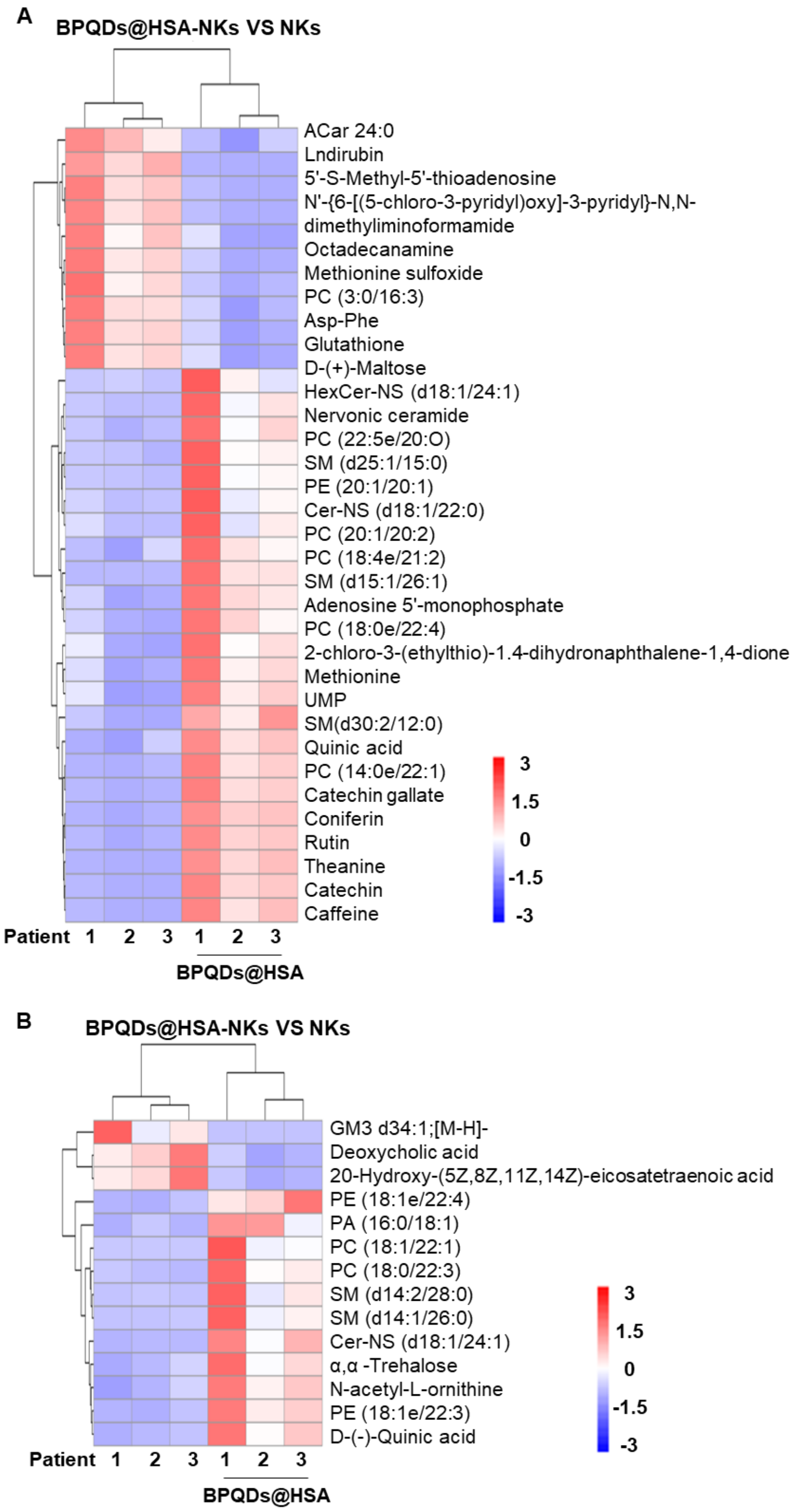

Figure S17. Heatmap of the differential metabolite under positive (**A**) and negative (**B**) ion mode in the three tumor patients-donated NK cells treated by BPQDs@HSA.

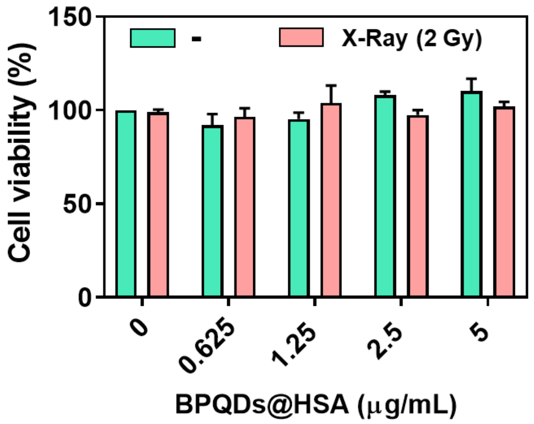

Figure S18. Cytotoxic effects of BPQDs@HSA on NK cells exposed with or without X-ray radiation (means ± S.D., n = 3).

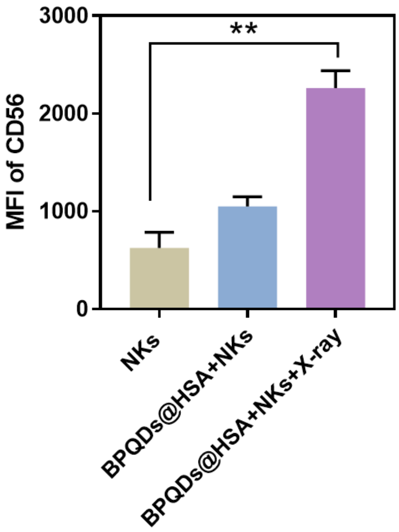

Figure S19. The mean fluorescence intensity (MFI) of CD56 in tumor sections of different treatment mice.

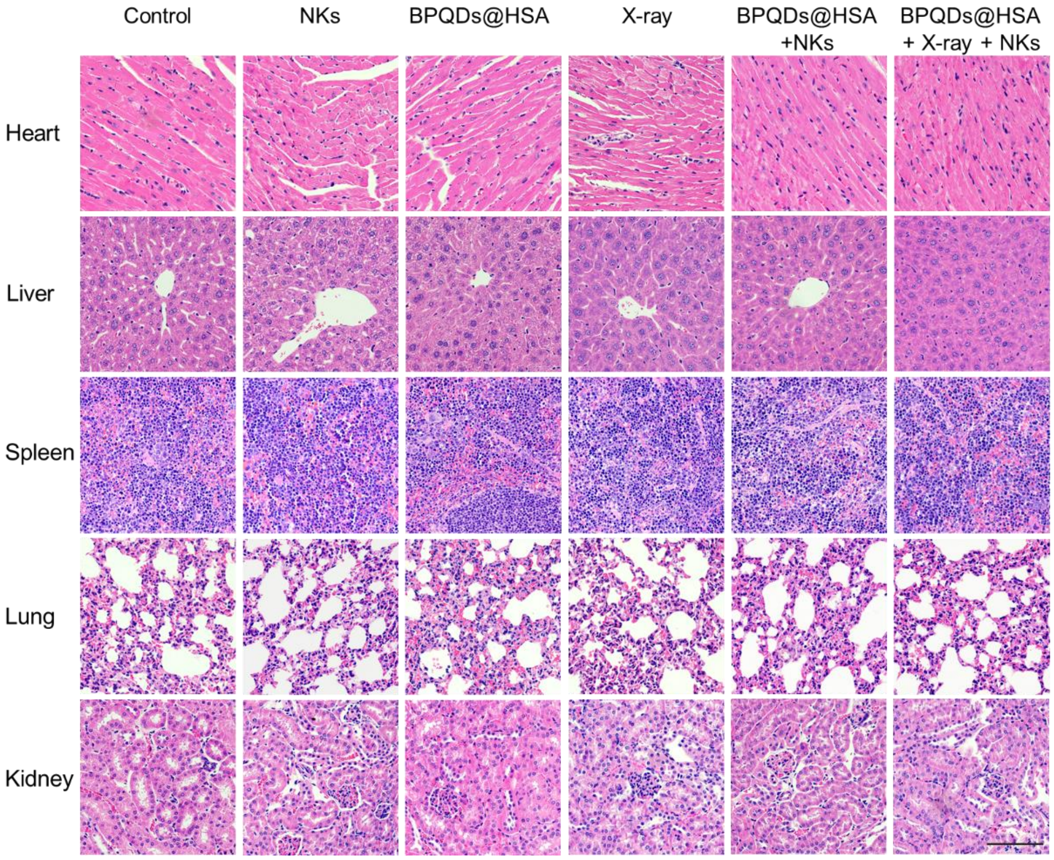

Figure S20. H&E staining of heart, liver, spleen, lung and kidney tissues in different treatment groups. Scale bar, 100 μm.

Table S1. The information of the volunteers.

Volunteers	Gender	Age	Cell ratio of $CD^{3-}CD^{56+}$ (%)	Cell ratio of $CD^{3+}CD^{56+}$ (%)
Patient 1	Male	64	92.82	1.26
Patient 2	Male	53	97.39	0.86
Patient 3	Male	58	93.13	1.22
Patient 4	Female	64	98.59	0.35
Patient 5	Male	61	96	1.71
Patient 6	Male	85	85.02	6.22
Patient 7	Male	64	80.33	6.14
Patient 8	Female	51	88.86	0.35
Patient 9	Male	34	81.76	2.53
Patient 10	Male	85	82.27	8.75

Table S2. Primer sequence for real-time PCR gene analysis.

Gen name	Primer direction	Primer pair
H-TLR1	forward 5'→3'	CCACGTTCCTAAAGACCTATCCC
	reverse 5'→3'	CCAAGTGCTTGAGGTTCACAG
H-TLR2	forward 5'→3'	TTATCCAGCACACGAATACACAG
	reverse 5'→3'	AGGCATCTGGTAGAGTCATCAA
H-TLR3	forward 5'→3'	TTGCCTTGTATCTACTTTTGGGG
	reverse 5'→3'	TCAACACTGTTATGTTTGTGGGT
H-TLR6	forward 5'→3'	TGAATGCAAAAACCCTTCACCT
	reverse 5'→3'	CCAAGTCGTTTCTATGTGGTTGA
H-TLR9	forward 5'→3'	CTGCCACATGACCATCGAG
	reverse 5'→3'	GGACAGGGATATGAGGGATTTGG
H-mTOR	forward 5'→3'	GCAGATTTGCCAACTATCTTCGG
	reverse 5'→3'	CAGCGGTAAAAGTGTCCCCTG
H-GSK3β	forward 5'→3'	TGGTCGCCATCAAGAAAGTATTG
	reverse 5'→3'	GCGTCTGTTTGGCTCGACTAT
GAPDH	forward 5'→3'	CCATGTTCGTCATGGGTGTGAACCA
	reverse 5'→3'	GCCAGTAGAGGCAGGGATGATGTTC
